# Catalytic surface radical in dye-decolorizing peroxidase: a computational, spectroscopic and site-directed mutagenesis study

**DOI:** 10.1042/BJ20141211

**Published:** 2015-02-20

**Authors:** Dolores Linde, Rebecca Pogni, Marina Cañellas, Fátima Lucas, Victor Guallar, Maria Camilla Baratto, Adalgisa Sinicropi, Verónica Sáez-Jiménez, Cristina Coscolín, Antonio Romero, Francisco Javier Medrano, Francisco J. Ruiz-Dueñas, Angel T. Martínez

**Affiliations:** *Centro de Investigaciones Biológicas, CSIC, Ramiro de Maeztu 9, E-28040 Madrid, Spain; †Department of Biotechnology, Chemistry and Pharmacy, University of Siena, I-53100, Siena, Italy; ‡Joint BSC-CRG-IRB Research Program in Computational Biology, Barcelona Supercomputing Center, Jordi Girona 29, E-08034 Barcelona, Spain; §Anaxomics Biotech, Balmes 89, E-08008 Barcelona, Spain; ║ICREA, Passeig Lluís Companys 23, E-08010 Barcelona, Spain

**Keywords:** catalytic protein radical, dye-decolorizing peroxidase, EPR spectroscopy, molecular docking, QM/MM, site-directed mutagenesis, ABTS, 2,2′-azinobis-(3-ethylbenzothiazoline-6-sulfonic acid), DMP, 2,6-dimethoxyphenol, DyP, dye-decolorizing peroxidase, hfcc, hyperfine coupling constant, LiP, lignin peroxidase, LRET, long-range electron transfer, MM, molecular mechanics, NBS, *N*-bromosuccinimide, PELE, Protein Energy Landscape Exploration, QM, quantum mechanics, RB5, Reactive Black 5, RB19, Reactive Blue 19, TNM, tetranitromethane, VA, veratryl alcohol, VP, versatile peroxidase, WT, wild-type

## Abstract

Dye-decolorizing peroxidase (DyP) of *Auricularia auricula-judae* has been expressed in *Escherichia coli* as a representative of a new DyP family, and subjected to mutagenic, spectroscopic, crystallographic and computational studies. The crystal structure of DyP shows a buried haem cofactor, and surface tryptophan and tyrosine residues potentially involved in long-range electron transfer from bulky dyes. Simulations using PELE (Protein Energy Landscape Exploration) software provided several binding-energy optima for the anthraquinone-type RB19 (Reactive Blue 19) near the above aromatic residues and the haem access-channel. Subsequent QM/MM (quantum mechanics/molecular mechanics) calculations showed a higher tendency of Trp-377 than other exposed haem-neighbouring residues to harbour a catalytic protein radical, and identified the electron-transfer pathway. The existence of such a radical in H_2_O_2_-activated DyP was shown by low-temperature EPR, being identified as a mixed tryptophanyl/tyrosyl radical in multifrequency experiments. The signal was dominated by the Trp-377 neutral radical contribution, which disappeared in the W377S variant, and included a tyrosyl contribution assigned to Tyr-337 after analysing the W377S spectra. Kinetics of substrate oxidation by DyP suggests the existence of high- and low-turnover sites. The high-turnover site for oxidation of RB19 (*k*_cat_> 200 s^−1^) and other DyP substrates was assigned to Trp-377 since it was absent from the W377S variant. The low-turnover site/s (RB19 *k*_cat_ ~20 s^−1^) could correspond to the haem access-channel, since activity was decreased when the haem channel was occluded by the G169L mutation. If a tyrosine residue is also involved, it will be different from Tyr-337 since all activities are largely unaffected in the Y337S variant.

## Abstract 2

We demonstrate that an exposed tryptophan is responsible for high-turnover oxidation by DyP, a representative of a new protein superfamily. Long-range electron transfer from surface tryptophan residues forming radicals appears as a general mechanism for peroxidase oxidation of bulky substrates.

## INTRODUCTION

DyPs (dye-decolorizing peroxidases) (EC 1.11.1.19) represent a new family of haem peroxidases widespread in bacteria, archaea, fungi and other micro-organisms [1–4]. Among those of fungal origin, the enzymes from *Bjerkandera adusta* [5–7] and *Auricularia auricula-judae* [8–10] have been crystallized and biochemically characterized as representative DyPs from two phylogenetically different basidiomycetes (in orders Polyporales and Agaricales respectively). The structures of bacterial DyPs were simultaneously solved [11–15]. *B. adusta* DyP was largely characterized as a recombinant protein [16,17], whereas *A. auricula-judae* DyP was isolated from fungal cultures [18]. The latter enzyme has recently been overexpressed in *Escherichia coli* as inclusion bodies, and a refolding protocol was optimized yielding a recombinant DyP with basically the same properties as those of wild-type DyP [19].

Xenobiotic anthraquinone-type dyes are the best-known substrates for DyPs. Among wood-rotting basidiomycetes, DyP genes are significantly more frequent in the sequenced genomes of white-rot (ligninolytic) than brown-rot species [20]. This fact and their reported capability to degrade non-phenolic lignin model dimers, although with much lower efficiency than white-rot fungal LiPs (lignin peroxidases) [8], suggest a possible contribution of fungal DyPs to lignin biodegradation. Similarly, lignin-degrading capabilities have been claimed for bacterial DyPs [21,22].

Both lignin polymer and substituted anthraquinone dyes, such as RB19 (Reactive Blue 19) (Supplementary Figure S1A), cannot easily access the buried haem cofactor in DyPs and other haem peroxidases. As an alternative for oxidation of these bulky substrates, LRET (long-range electron transfer) from radical-forming aromatic residues at the DyP surface has been suggested [9,23]. Surface residues at the origin of LRET routes were first reported in *Phanerochaete chrysosporium* LiP [24] and *Pleurotus eryngii* VP (versatile peroxidase) [25], and later identified in the sequences of many putative LiPs and VPs from genomes of lignin-degrading white-rot basidiomycetes [20].

Computational analyses can help to explain these LRET processes, requiring, however, the combination of different levels of theory [26]. Long-timescale processes, such as substrate binding, can only be accomplished through MM (molecular mechanics) methods, whereas electron transfer requires QM (quantum mechanics)-based methods, such as QM/MM [27]. The combination of these techniques was shown to be a successful approach in the study of oxidation and electron-transfer processes in haem proteins [28].

In the present study, we expressed *A. auricula-judae* DyP in *E. coli*, solved the crystal structure of the recombinant enzyme and several site-directed variants, and used PELE (Protein Energy Landscape Exploration) [29] to describe the binding of its typical substrate RB19. Subsequent QM/MM analyses of binding sites indicate a preference for substrate oxidation at an exposed tryptophan residue, and identified the LRET pathway to haem. Simultaneously, a mixed tryptophanyl/tyrosyl radical was detected by EPR spectroscopy of the H_2_O_2_-activated WT (wild-type) DyP. A combined multifrequency EPR and computational approach, together with site-directed mutagenesis studies, enabled the identification of both protein radical contributions. Moreover, we associated a high-turnover site in DyP to the presence of a tryptophanyl radical, in agreement with the QM/MM predictions. In this way, a multidisciplinary evaluation of the role of protein radicals in DyP catalysis is provided.

## MATERIALS AND METHODS

### Chemicals

Among DyP substrates, RB19, DMP (2,6-dimethoxyphenol), RB5 (Reactive Black 5) and VA (veratryl alcohol) were from Sigma–Aldrich, and ABTS [2,2′-azinobis-(3-ethylbenzothiazoline-6-sulfonic acid)] was from Boehringer Mannheim (see Supplementary Figures S1A–S1E respectively and the Supplementary Methods for other chemicals).

### DyP production, activation and purification

The DNA sequence coding mature DyP-I from *A. auricula-judae* (GenBank® accession number JQ650250) [18] was synthesized (ATG:biosynthetics), expressed in *E. coli*, activated *in vitro*, and purified as described in [19] (see the Supplementary Methods for details).

### Site-directed mutagenesis and chemical modification of DyP

Simple DyP variants were produced by PCR using the pET23a-DyPI vector harbouring the mature protein-coding sequence of *A. auricula-judae* DyP as a template. For each mutation, direct and reverse primers were designed. For double (or triple) mutations, the mutated vector for the first (or second) mutation was used as template. The pET23a-DyPI plasmids containing the mutations were digested with endonuclease DpnI and transformed into *E. coli* DH5α cells for propagation.

Tryptophan and tyrosine residues in 3 μM WT DyP and the W377S variant were also chemically modified using up to 0.3 mM NBS (*N*-bromosuccinimide) and up to 40 mM TNM (tetranitromethane) (including 2.6% ethanol) respectively [30]. Chemically modified enzymes were used for estimation of residual activity on RB19 (180 μM), DMP (7.5 mM), RB5 (15 μM) and ABTS (1.25 mM) (see the Supplementary Methods for PCR primers and conditions, and details on chemical modification).

### Crystallization, data collection and refinement

Crystallization of WT DyP and five site-directed variants was optimized by the sitting-drop vapour-diffusion method. Crystals of WT DyP were obtained in 32.5% PEG 4000, and those of all the variants were obtained in PEG 2000 MME (30–35%). X-ray diffraction intensities were collected at the SOLEIL (Gyf-sur-Yvette, France) and ALBA (Barcelona, Spain) synchrotrons. The structure of WT DyP and its variants were solved by molecular replacement (see the Supplementary Methods for details; collection, refinement and final statistics are in Supplementary Table S1). Some of the structures did not show electron density for the first two or three residues at the N-terminus, but the whole sequence could be solved for two of them (PDB codes 4W7K and 4W7L). In contrast, the C-terminal region showed good electron density for all of the structures.

### Enzyme kinetics

Steady-state kinetic constants were determined from absorbance increases during oxidation of DMP, ABTS and VA at pH 3 (pH 2.5 for VA) measured using a Thermo Spectronic UV–visible spectrophotometer. Absorbance decreases were followed for RB5 and RB19 oxidation (assayed at pH 3 and pH 3.5 respectively) using the same equipment. Eventual changes of enzyme molecular mass after turnover were investigated by MALDI–TOF (see the Supplementary Methods for details). Plotting and analysis of kinetic curves were carried out with SigmaPlot (version 11.0). Apparent affinity, turnover number and catalytic efficiency were estimated by non-linear least-squares fitting to the Michaelis–Menten model. The catalytic efficiency for VA was estimated by linear regression, since no saturation was attained. Calculation of two sets of kinetic constants was performed by adjusting to the Michaelis–Menten model the data from 0.2–10 μM RB19, 4–60 μM DMP, and 0.2–7 μM ABTS, separately from those of 50–270 μM RB19, 200–8000 μM DMP and 30–5000 μM ABTS.

### Computational analyses: PELE, MD and QM/MM calculations

The starting structure (based on 4W7J) was prepared at pH 3.5, the optimal pH for RB19 oxidation, by adjusting the protonation state of ionizable residues. Histidine residues were double-protonated, except for His-304 (δ-protonated) and His-115 (ε-protonated), and several aspartic acids (residues 8, 12, 84, 129, 189, 246 and 270) and glutamic acids (residues 158, 220, 225 and 432) were kept in their acidic form. The RB19 atomic charges were derived from QM calculations (see the Supplementary Methods for details of system preparation). Then, RB19 was placed manually in 20 initial random positions on the protein surface and the protein–ligand conformational space was explored with PELE [29]. Results shown are based on 160 independent 48-h PELE simulations. Enhanced local sampling on Trp-377 was obtained with a 5 ns MD simulation allowing us to investigate the effect of solvent and charge fluctuations on the oxidative tendency of Trp-377 and RB19. QM/MM calculations were performed with QSite 5.7 (Schrödinger). Trp-377 LRET pathway calculations were performed with the QM/MM e-pathway approach [32] with His-304–Arg-311, Leu-323–Ala-325, Leu-373–Gln-375 and Asp-395 in the quantum region.

### EPR spectroscopy and parameter calculations

CW (continuous wave) X-band (9.8 GHz) and W-band (94.17 GHz) experiments were recorded on Bruker Elexsys spectrometers E500 and E600 respectively (see the Supplementary Methods for details). DyP (0.1 μM) activation was carried out using an enzyme/H_2_O_2_ molar ratio of 1:10 in tartrate, pH 3. H_2_O_2_ addition was done directly in the EPR tube for the X-band measurements, and the reaction time before freezing was less than 10 s. For the W-band measurements, the H_2_O_2_ addition was done before filling the EPR tube resulting in a longer freezing time for the sample. Spectra simulations were performed by the Easyspin 4.5.5 package using the ‘Pepper’ function [33]. Preparatory force field calculations were performed before QM/MM estimation of EPR magnetic parameters. The QM/MM calculations were performed with the MOLCAS 7.4 package [34] coupled with a modified version of the MM package Tinker 4.2. EPR magnetic parameters - g-tensors, hfcc (hyperfine coupling constant) values and Mulliken spin densities - were computed via single-point calculations on the optimized structures using the ORCA2.9 package (F. Neese, University of Bonn, Bonn, Germany). Details of the protocols used to compute the EPR parameters are reported by Bernini et al. [36,37]

## RESULTS AND DISCUSSION

### Molecular structure: general fold and exposed aromatic residues

The crystal structure of *A. auricula-judae* WT DyP expressed in *E. coli* was solved at 1.79 Å (1 Å=0.1 nm) resolution (PDB 4W7J), together with those of the Y147S, D168N, W377S, Y147S/W377S and Y147S/G169L/W377S variants (PDB codes 4W7K, 4W7L, 4W7M, 4W7N and 4W7O) solved at 1.05–1.40 Å resolution (Supplementary Table S1). The recombinant DyP is similar (0.48 Å RMSD, 1776 atoms) to the enzyme isolated from a fungal culture (PDB 4AU9). Moreover, most of the variants show crystal structures largely superimposable with that of WT DyP, except for the mutated residues.

**Figure 1:**
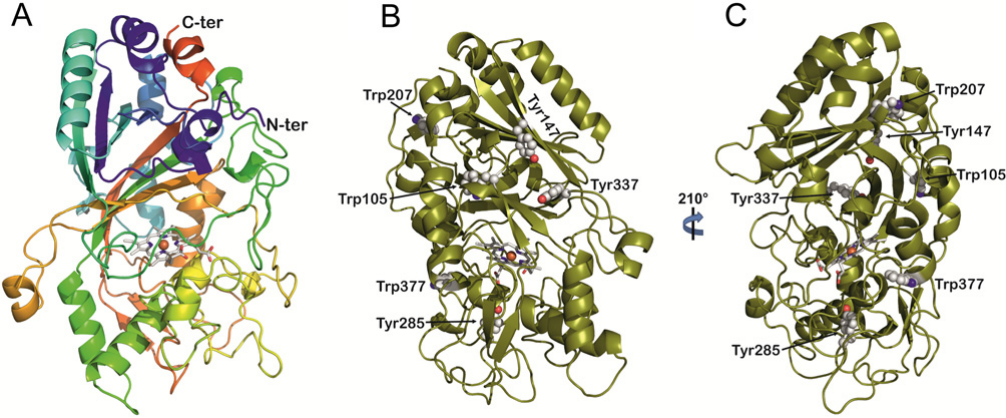
Folding of *A. auricula-judae* DyP and location of exposed aromatic residues (**A**) General folding constituted by two domains, each of them including two large β-sheets and two or three helices, with the haem cofactor in the upper part of the lower domain [cartoon coloured from the N- to the C-terminus, with the haem shown as CPK (Corey–Pauling–Koltun) sticks]. (**B** and **C**) Location of exposed Trp-105, Tyr-147, Trp-207, Tyr-285, Tyr-337 and Trp-377 (as CPK spheres) in two different orientations of the DyP molecule (cartoon with the haem shown as CPK sticks). From PDB code 4W7J.

The DyP structure is formed by two domains, each of them including an antiparallel four-stranded large β-sheet and two or three helices resulting in a ferredoxin-like fold (plus two additional β-strands) (Figure 1A). The C-terminal region also includes two small additional helices extending into the N-terminal domain. In spite of the obvious similarity between the two domains, only the C-terminal domain harbours a haem cofactor. His-304 (N_ε_) acts as the fifth ligand of the haem iron, with Asp-395 at 2.66 Å. At the opposite side of the haem, Asp-168 and Arg-332 occupy neighbouring positions, suggesting a contribution to the haem reaction with H_2_O_2_. A single cysteine residue (Cys-299) is present in the C-terminal domain of DyP. The above structural characteristics of the *A. auricula-judae* and other DyPs indicate a common origin with the other members of the CDE superfamily [38] comprising chlorite dismutase [39], DyP and *E. coli* EfeB proteins [13]. Therefore similarities in the haem pocket architecture with the superfamily of classical plant/fungal/prokaryotic peroxidases [40] result from adaptive convergence to provide similar reactivity properties to the haem cofactor (see the Supplementary Results and Discussion).

Near the confluence of the two domains, a channel provides access to the haem cofactor that, due to its location in DyPs, connects to the top of the haem (Supplementary Figure S2). H_2_O_2_ will enter through this channel to activate the enzyme, forming compound I. However, direct oxidation of typical DyP substrates, such as RB19 and other bulky dyes, by the activated haem is not possible due to the narrow opening of the channel. Therefore LRET appears as a feasible alternative. This is in agreement with the high number of aromatic residues in the *A. auricula-judae* DyP sequence, including seven tyrosine residues and four tryptophan residues. The exposed nature of six of them (Figures 1B and 1C) suggests participation in LRET oxidation by forming reactive radicals at the protein surface. This is reminiscent of that found in ligninolytic peroxidases (LiPs and VPs) where the bulky lignin polymer is oxidized by LRET from an exposed protein radical [41–45]. Interestingly, ligninolytic peroxidases have none or only a few tyrosine residues in their sequences, a fact that has been considered as a protection against oxidative inactivation [46]. One remarkable exception is the *Trametopsis cervina* LiP that has a tyrosine residue involved in catalysis [41]. In the molecular models of other LiPs and VPs isolated from fungi or identified from genomes [20], an exposed tryptophan acts as the oxidation site for high-redox-potential aromatics, dyes and polymeric lignin via LRET [47].

### Computational simulations: substrate binding (PELE) and LRET pathways (QM/MM)

**Figure 2:**
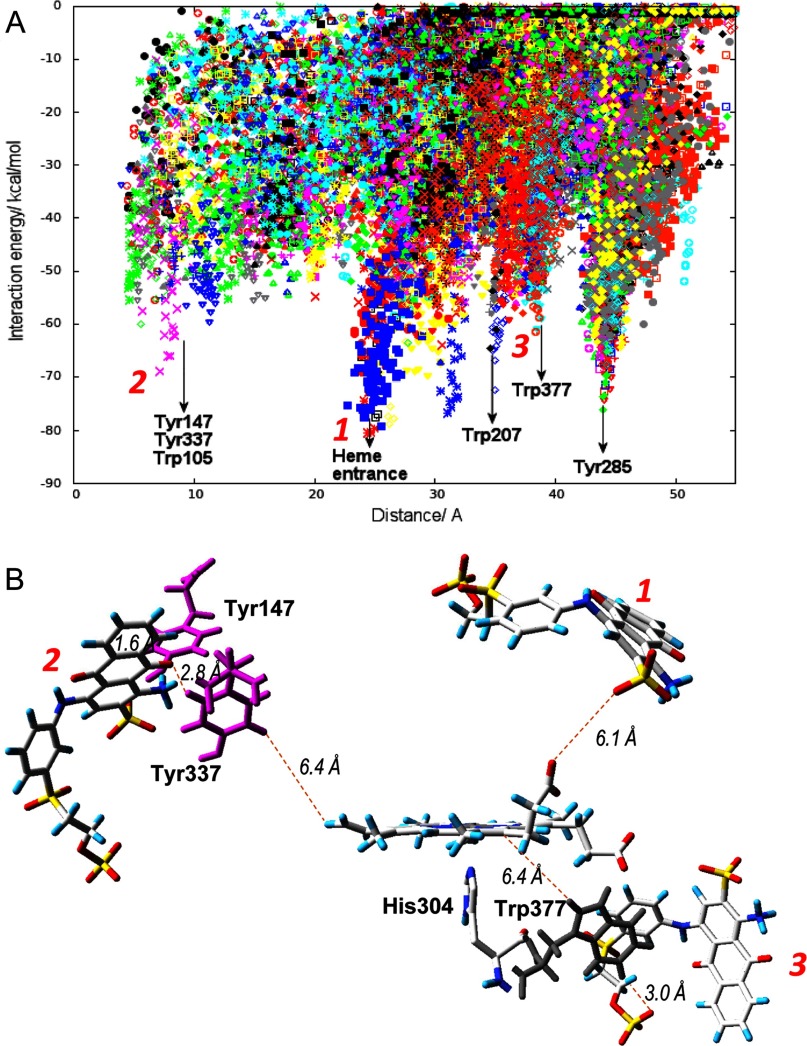
Substrate exploration on the DyP surface (**A**) Local minima identified in the PELE [29] simulations of RB19 diffusion on the recombinant DyP crystal structure (PDB code 4W7J) showing interaction energy against distance to Tyr-147 (taken as a reference residue). The presence of RB19 in the vicinity of different surface residues and the haem-access channel is indicated. (**B**) Distances between the closest positions of RB19 (magenta sticks) with respect to haem (1), Tyr-147/Tyr-337 (2), and Trp-377 (3) shown by PELE (**A**), and between the above residues and the haem cofactor (the distances are measured including hydrogen atoms). RB19 is shown as CPK (Corey–Pauling–Koltun) sticks, Tyr-147/Tyr-337 as magenta sticks and Trp-377 as grey sticks.

To identify the possible substrate binding site(s) in *A. auricula-judae* DyP, we performed 160 PELE [29] non-biased simulations, where the typical DyP substrate RB19 was free to explore the structure of the recombinant enzyme. These simulations (Figure 2A) show that RB19 encounters several favourable docking positions on the protein surface, with local minima close to Trp-105, Tyr-147/Tyr-337, Trp-207, Tyr-285 and Trp-377 sites, as well as in the haem channel entrance. Among them, Tyr-147, Tyr337 and Trp-377 are solvent-exposed, but Trp-105 and Tyr-285 are buried into the protein (Supplementary Figure S3) making their interaction with the dye substrate more difficult. It is important to keep in mind that, whereas simulations were performed with only one substrate molecule (see Supplementary Movie S1 for an example of the exploration), under *in vitro* reaction conditions (with a large excess of substrate) multiple local minima might be populated to different extents. To address computationally which minimum will oxidize the substrate, we used QM/MM studies.

**Figure 3:**
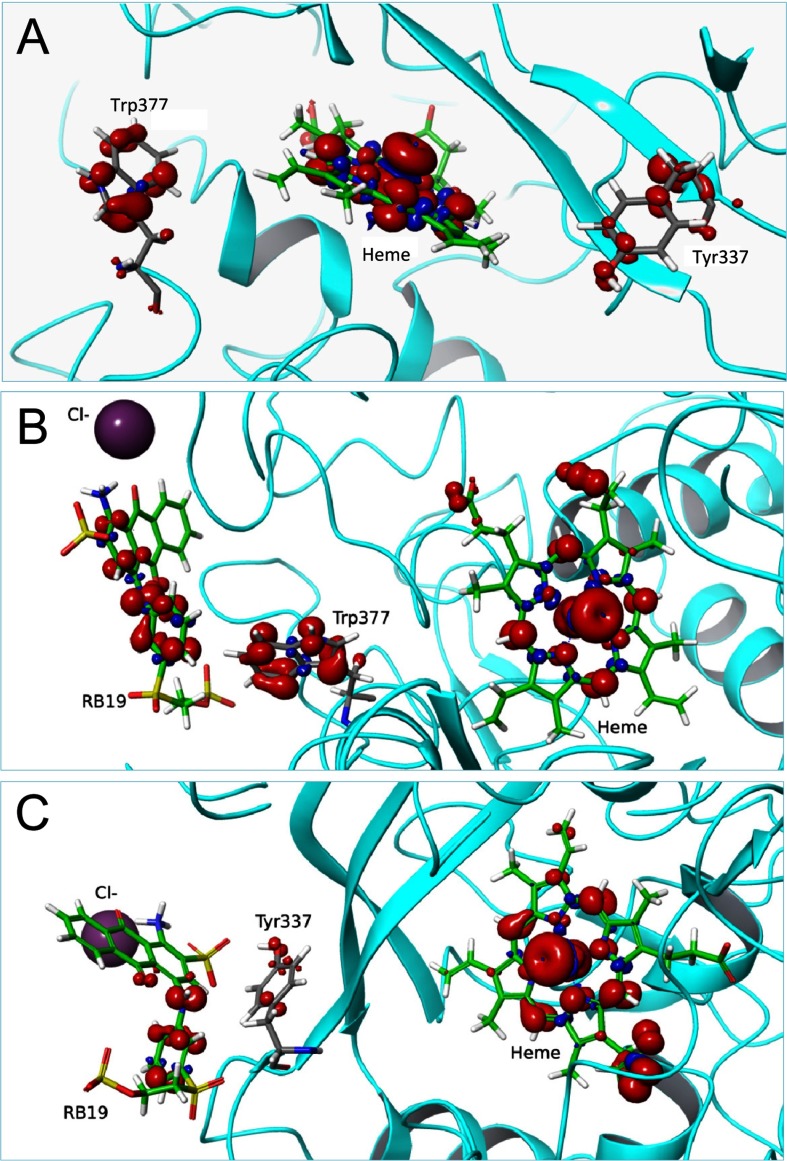
QM/MM electron spin distribution on Trp-377 and Tyr-337 and RB19 substrate (**A**) Total spin density when including Trp-377, Tyr-337 and haem compound I in the quantum region (in the absence of RB19). (**B** and **C**) Total spin density when, in addition to RB19 and compound I, the quantum region includes Trp-377 or Tyr-337 respectively. From PDB code 4W7J after 5 ns MD (**A**) and selected snapshots from two energy minima showing RB19 near Trp-377 and Tyr-337, during PELE [29] diffusion in Figure 2 (**B** and **C** respectively).

First, we performed a simple QM/MM pairwise comparison between Trp-377 and the other residues identified in the protein exploration with PELE, by including only the two selected residues in the quantum region, subtracting one electron and computing the spin density. There is a clear preference for Trp-377 to be oxidized over Trp-105, Tyr-285 and Tyr-337 (Supplementary Table S2). A comparison of Trp-207 and Trp-377 suggests that both residues could be oxidized. However, electron coupling exponentially decays with donor–acceptor distance and we can therefore exclude Trp-207 due to its large distance from the haem iron (Supplementary Figure S3). To investigate further the oxidation of Trp-377 and Tyr-337 by compound I, we performed new calculations where, in addition to these two residues, the haem was modelled as compound I and included in the quantum region (Figure 3A). The total spin density at Trp-377 shows its preferential oxidation by compound I, validating the previous pairwise analysis, although some density was also observed at Tyr-337.

As a final step, substrate oxidation was investigated in new calculations where we added to the quantum region RB19 at the best PELE position for each of the two residues. Figure 3(B) shows the total spin density for a structure including the dye, the surface Trp-377 and the haem cofactor. Spin density depends on the local electrostatic environment (it was previously found that Trp-377 oxidation was 40% improved in the presence of a neighbouring Cl^−^ ion). In agreement with these results, the presence of anionic RB19 enhances Trp-377 oxidation. Similarly, we investigated the possibility of RB19 oxidation in the Tyr-337 site (Figure 3C), where spin density in the tyrosine and substrate are observed. Nevertheless, the most favourable residue for substrate oxidation on the protein surface is Trp-377 and so even though other surface residues may act as potential oxidizing sites, these would have a minor participation in catalysis.

**Figure 4:**
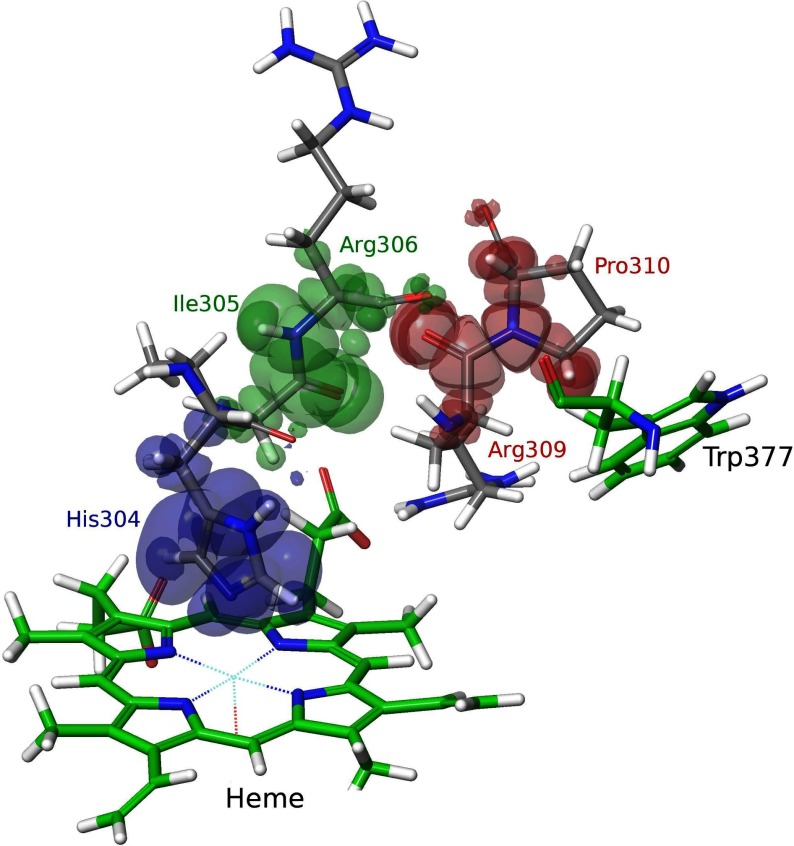
Electron transfer pathway from DyP Trp-377 to haem The electron transfer pathway was obtained after three iterations of the QM/MM e-pathway approach [32] with a total of 15 residues (His-304–Arg-311, Leu-323–Ala-325, Leu-373–Gln-375 and Asp-395) included in the quantum region. Each iteration identifies the residue(s) with the highest affinity for the electron, and is shown in a different colour. The mapped route includes Pro-310 and Arg-309, followed by Arg-306, Ile-305 and His-304, as shown by the electron spin distribution.

Finally, using QM/MM methods, we were able to map the important residues along the LRET pathway from WT DyP Trp-377 to the haem (Figure 4). This pathway would include a first 3.0 Å electron transfer between the Trp-377 side chain and the Pro-310/Arg-309 backbone, and a second one (2.9 Å) between the Arg-309 and Arg-306 carbonyls, followed by the Arg-306 to His-304 backbone to reach the haem iron (at only 2.2 Å from the His-304 side chain). The path depicted by the QM/MM e-pathway approach [32] used in the present study is more precise than that previously predicted for the same residue using simpler geometric methods [23].

### Catalytic protein radicals: EPR detection in WT DyP and mutated variants

**Figure 5:**
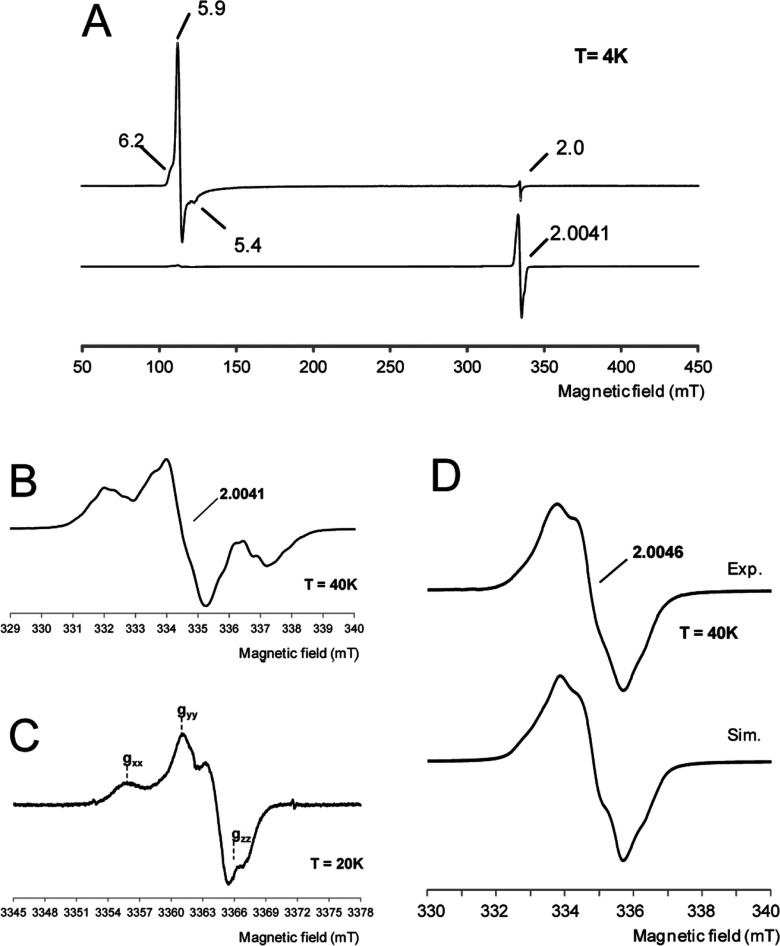
EPR spectra of WT DyP and its W377S variant (**A**) X-band EPR spectra of WT DyP at pH 3 before (top) and after (bottom) the addition of H_2_O_2_ (and rapid freezing). Experimental conditions: *v*=9.39 GHz, 0.2 mW microwave power, 0.4 mT modulation amplitude. (**B** and **C**) Narrow scan X-band (*v*=9.39 GHz) and W-band (*v*=94.29 GHz) respectively of the radical species. The positions of the three g-tensor components of the tyrosyl contribution are indicated. X-band experimental conditions: *v*=9.38 GHz, 1 mW microwave power and 0.05 mT modulation amplitude; W-band experimental conditions: *v*=94.29 GHz, 0.05 mW microwave power and 0.1 mT modulation amplitude. (**D**) X-band EPR spectrum of the radical intermediate formed in the W377S variant paired with its better simulation (Sim) (see magnetic parameters in Supplementary Table S3). Experimental conditions: *v*=9.39 GHz, 1 mW microwave power, 0.2 mT modulation amplitude.

The EPR spectrum of the WT DyP resting state (Figure 5A, top) shows a ferric species prevalently in its axial high spin state (*g*_⊥_≈ 6 and *g*_∥_=2.0). After adding 10 eq. of H_2_O_2_, a strong decrease in the ferric signal, and appearance of an intense protein radical signal are evident (Figure 5A, bottom) (the electronic absorption spectra of WT DyP, and the EPR and electronic absorption spectra of the D168N and R332L variants are described in the Supplementary Results and Supplementary Figures S4 and S5 respectively). The yield of the radical observed in the spectrum of the H_2_O_2_-activated WT DyP is estimated as 0.58 spin/haem. Expansion of the EPR spectrum (Figure 5B) shows a protein radical signal centred at *g*=2.0041(1) with two low and high field components, and several sub-splittings. The overall lineshape suggests the presence of two different radical contributions, with the low field side of the spectrum less structured than the high field side, and an intense central line. To identify the radical contributions based on their different anisotropy [48–50], high-frequency EPR spectra were recorded. The narrow scan of the 94 GHz EPR spectrum of H_2_O_2_-activated WT DyP shows the contributions from two radicals with different g-tensor anisotropy (Figure 5C). For a tryptophanyl radical, 94 GHz EPR (3.3 T) still does not represent the high-field limit where the three g-tensor components are separated, whereas for tyrosyl radicals g-tensor components are well separated at 94 GHz (Figure 5C) and enabled site assignment of the protein radical.

The proof for Trp-377 being involved in the mixed protein radical was provided by the W377S variant. In the high-resolution narrow scan of its 9 GHz EPR spectrum (Figure 5D, top), the tryptophanyl contribution observed for WT DyP (Figure 5B) completely disappeared (the spectrum of the W377S/Y147S variant, not shown, being nearly superimposable). The W377S spectrum (Figure 5D, top) shows a single line with hyperfine resolution at *g*=2.0046(2). Simulation of this spectrum at X-band (Figure 5D, bottom) confirmed that it corresponds to a tyrosine phenoxyl radical. This identification was obtained by taking into account the experimental g-tensor component, *g*_xx_=2.0075 from the high-field EPR spectrum, and the hf-tensor data from the simulated 9 GHz EPR spectrum. The β-protons’ hfccs agree with the computed constants for Tyr-337 (Supplementary Table S3). Therefore this residue would be responsible for the tyrosyl contribution observed in WT DyP, mixed with the main Trp-377 radical contribution.

It had been claimed that Tyr-337 was responsible for substrate oxidation by *A. auricula-judae* DyP based on spin trapping and TNM modification (see below) results [9]. Although their redox potential is affected by pH and residue environment [51], tyrosyl radicals are less reactive than tryptophanyl radicals, as shown for *P. eryngii* VP whose W164Y variant lost activity on RB5 and VA [42]. In the present study, we confirm that Tyr-337 forms a radical during H_2_O_2_ activation of *A. auricula-judae* DyP, but this radical represents a relatively minor contribution of the mixed tryptophanyl/tyrosyl radical signal detected (and Tyr-337 is not catalytically relevant, as discussed below) in agreement with QM/MM spin calculations. Formation of a protein radical was also suggested for *Rhodococcus jostii* DyP [14]. Tryptophanyl and tyrosyl radicals have been identified in different redox enzymes [41,47,52], and it has been suggested that both could be involved in substrate oxidation by DyPs [9,10]. In the present study, we have detected directly, for the first time, a protein radical in a DyP, whose tryptophanyl and tyrosyl contributions were identified by a combined EPR multifrequency and computational approach.

### Catalytic properties after chemical modification and site-directed mutagenesis

First, the effect of pH on DyP activity was analysed (Supplementary Figure S6) and the optimal values (pH 3.5 for RB19, pH 2.5 for VA, and pH 3.0 for DMP, ABTS and RB5) were used in subsequent studies. Acidic pH optima were already reported for *A. auricula-judae* DyP [19], and are also typical of lignin-degrading peroxidases (LiP and VP) [46,53]. It is interesting that a delay period was not observed in oxidation reactions with WT DyP, and its W377S variant described below, which showed identical reaction traces and MALDI–TOF molecular masses with/without treatment with VA and H_2_O_2_ (Supplementary Figures S7A and S7B). This permits us to rule out in DyP an activation mechanism similar to that of *T. cervina* LiP, which enabled a tyrosine residue to oxidize high-redox-potential substrates after forming a reactive adduct with VA [54].

**Figure 6:**
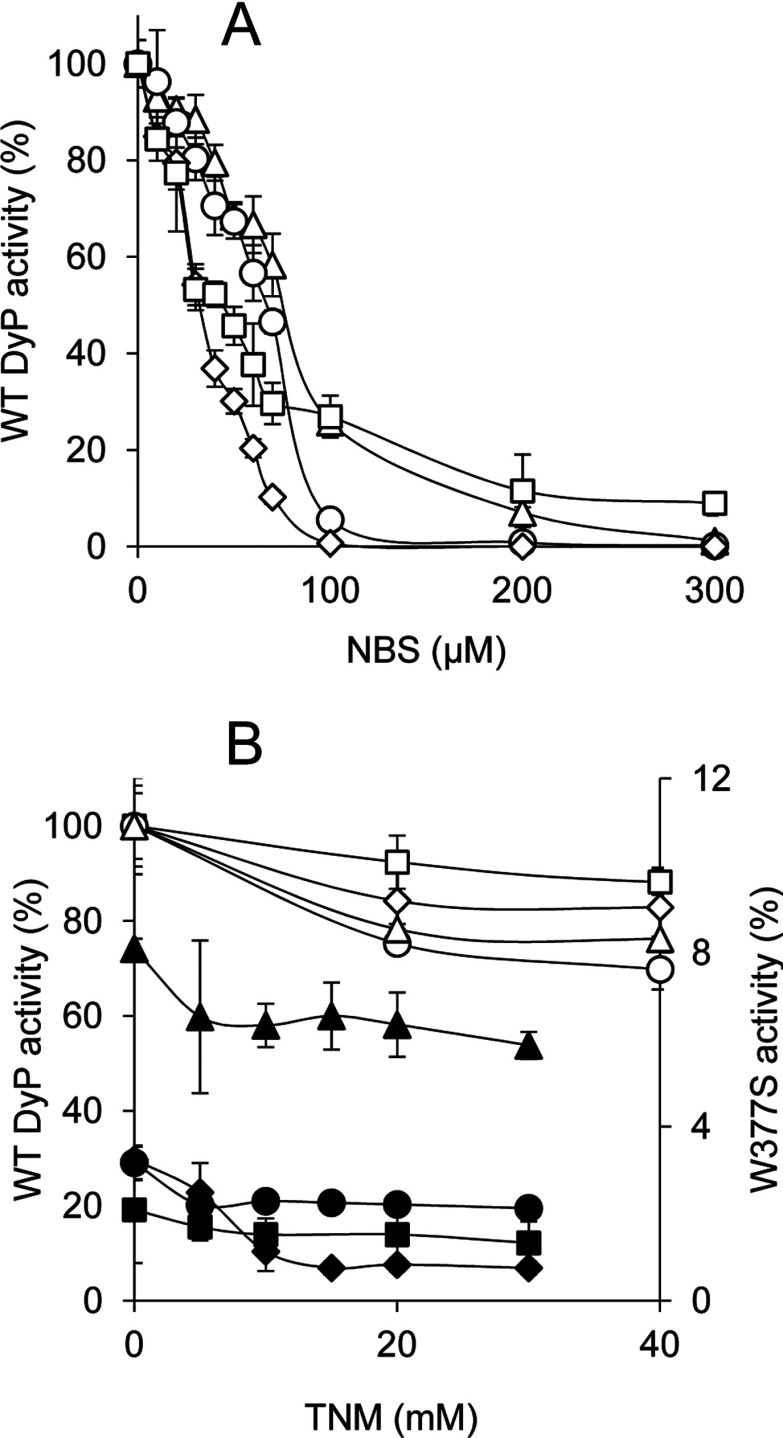
Chemical modification of tryptophan and tyrosine residues in WT DyP and its W377S variant (**A**) Residual activities of 3 μM WT DyP treated with increasing NBS concentrations (in 50 mM acetate, pH 4) for modification of tryptophan residues. (**B**) Residual activities of 3 μM WT DyP and W377S variant (white and black symbols respectively) treated with increasing TNM concentrations (in 50 mM Tris/HCl, pH 7, with 2.6% ethanol) for modification of tyrosine residues. The residual activities of the WT DyP and the W377S variant in (**A**) and (**B**) were monitored for oxidation of 180 μM RB19 (diamonds), 7.5 mM DMP (circles), 15 μM RB5 (squares) and 1.25 mM ABTS (triangles), and referred to activities of untreated WT DyP (taken as 100%).

In a first approach for residue modification, *A. auricula-judae* DyP (3 μM) was treated with NBS, which oxidizes the tryptophan ring to oxindole [55], and TNM, which nitrates the phenolic ring of tyrosine [56]. Near 90% activity on the four substrates assayed was removed when NBS (up to 300 μM) was used (Figure 6A). This confirms that most DyP activity is associated with tryptophan residue(s), whereas less than 15% would be due to a different site(s). Similar TNM concentrations did not affect DyP activity, but a partial decrease was observed when a 200-fold higher concentration was used (Figure 6B, white symbols). This indicates that some tyrosine residue(s) contribute (directly or indirectly) to substrate oxidation by DyP, although to a lower extent than the tryptophan residue(s). Additional experiments analysed the effect of chemically modifying tyrosine residues on the very-low-activity W377S variant described below (Figure 6B, black symbols). Although activity decrease with some substrates (such as RB19) was observed, the activity with others (such as ABTS) was only slightly reduced, indicating that, in these cases, residues other than tyrosine residues (or maybe the haem cofactor) are involved.

**Figure 7:**
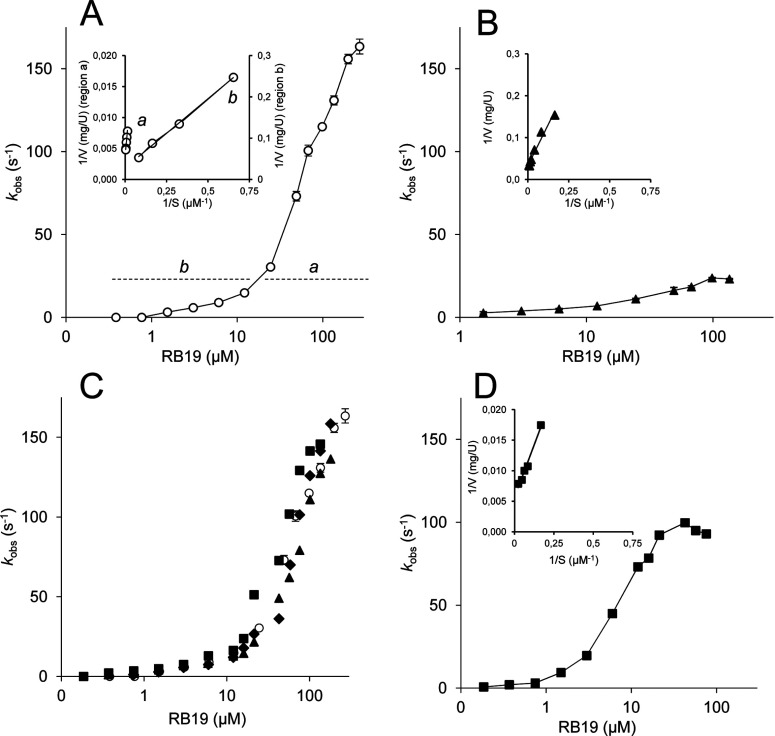
Kinetics for RB19 oxidation by WT DyP and different variants (**A**) WT DyP biphasic kinetics enabling calculation of two sets of constants in the 0.2–10 μM (*b*) and 50–270 μM (*a*) ranges (inset with Lineweaver–Burk inverse representation for the high, *a*, and low, *b*, turnover sites). (**B**) Simple kinetics yielding a single set of constants (inset, inverse representation) for the W377S variant. (**C**) Results from Y147S (squares), Y337S (diamonds) and Y147F/Y337F (triangles) variants yielding kinetic curves superimposable with that of WT DyP (circles). (**D**) Simple kinetics yielding a single set of constants (inset, inverse representation) for the G169L variant. A substrate concentration logarithmic scale is used in the main plots.

An interesting initial observation, when analysing the kinetics of DyP oxidations, was the bimodal curves obtained for most substrates (Figure 7A, regions *a* and *b*). Similar sigmoidal kinetic curves were recently reported for DyP [19], and previously for *P. eryngii* VP [57]. Such curves enable calculation of two set of constants (Table 1) and reveal the existence of, at least, two oxidation sites for the same substrate. The *a* and *b* sites in *A. auricula-judae* DyP are characterized by a high turnover (*k*_cat_) with low apparent affinity (as shown by the *K*_m_ values), and a low turnover with high apparent affinity respectively. The kinetic constants obtained show that the *A. auricula-judae* DyP is very efficient at oxidizing RB19 and ABTS dyes. However, it has lower catalytic efficiencies on DMP and RB5, and extremely low activity on VA. Finally, no Mn^2+^ oxidation activity was observed, as reported for bacterial DyPs [14,15].

**Table 1:** Steady-state kinetic constants of WT DyP and five site-directed variants *K*_m_ (*μ*M), *k*_cat_ (s^−1^), and *k*_cat_/*K*_m_ (s^−1^·mM^−1^) of WT DyP and its Trp-377, Tyr-147, Tyr-337 and Gly-169 variants oxidizing RB19, DMP, ABTS, RB5 and VA (including two sets of constants for the first four substrates corresponding to a high-turnover and a low-turnover site). Means and 95% confidence limits from reactions at 25°C in 0.1 M tartrate, pH 3 (pH 3.5 for RB19 and pH 2.5 for VA) using 0.1 mM H_2_O_2_ and 10 nM enzyme (100 nM for VA oxidation), are shown (when 1 mM H_2_O_2_ concentration was used, lower catalytic efficiencies were obtained, although the *k*_cat_ values often increased). NS, no saturation preventing kinetic constant estimation.

		WT DyP	W377S	Y147S	Y337S	Y147F/Y337F	G169L
RB19 (high turnover)	*K*_m_	90±10	–	95±24	83±3	130±30	10±3
	*k*_cat_	224±10	0	175±24	240±47	220±16	106±9
	*k*_cat_/*K*_m_	2460±180	–	1860±300	2700±400	1680±170	12400±3000
RB19 (low turnover)	*K*_m_	14.0±2.0	3.9±0.6	6.4±0.8	7.4±2.3	5.8±0.8	–
	*k*_cat_	32.0±3.0	8.9±0.6	26.0±6.1	26.0±1.9	18.0±1.9	0
	*k*_cat_/*K*_m_	2230±200	2240±240	4070±640	3370±350	3200±200	–
DMP (high turnover)	*K*_m_	703±61	–	763±70	2840±300	4720±460	353±41
	*k*_cat_	120±3	0	71±2	228±18	216±7	88±3
	*k*_cat_/*K*_m_	200±18	–	93±7	80±1.1	46±3	200±21
DMP (low turnover)	*K*_m_	6.0±0.5	3560±250	38.0±0.4	3.1±0.6	0.7±0.1	6.0±0.4
	*k*_cat_	8.0±0.2	6.4±0.1	15.0±0.9	8.4±0.3	3.9±0.1	9.9±0.2
	*k*_cat_/*K*_m_	1350±100	1.8±0.1	397±41	2730±520	5900±1000	1600±70
ABTS (high turnover)	*K*_m_	121±7	2750±470	366±30	239±29	173±21	25±2
	*k*_cat_	224±3	171±15	311±13	288±10	286±7.2	96±2
	*k*_cat_/*K*_m_	1850±94	62±1	850±80	1200±70	1650±180	3770±270
ABTS (low turnover)	*K*_m_	3.1±1.1	30.0±0.5	20.0±6.0	2.0±0.2	17.0±3.6	–
	*k*_cat_	7.4±1.4	14.0±1.0	21.0±4.0	2.3±0.3	21.0±6.0	0
	*k*_cat_/*K*_m_	2370±420	472±61	1010±100	1100±100	1260±120	–
RB5	*K*_m_	15.6±2.0	10.6±1.0	15.9±1.7	13.4±1.7	39.0±6.9	5.8±0.6
	*k*_cat_	4.8±0.2	0.70±0.02	8.3±0.6	5.2±0.3	9.1±2.6	1.2±0.1
	*k*_cat_/*K*_m_	310±20	68±4	525±230	400±29	233±20	217±13
VA	*K*_m_	NS	7360±1270	NS	NS	NS	NS
	*k*_cat_	–	0.23±0.01	–	–	–	–
	*k*_cat_/*K*_m_	0.096±0.002	0.032±0.003	0.101±0.005	0.079±0.011	0.095±0.002	0.130±0.006

Then, the exposed aromatic residues identified in PELE and QM/MM simulations were substituted to verify their eventual involvement in catalysis (information on the effect of mutating haem pocket residues is provided in the Supplementary Results). Aromatic residues were changed to serine in the simple W377S, Y147S and Y337S variants (the latter with low refolding yield). The double variant combining the last two mutations could not be refolded, but Y147F/Y337F could be obtained. Since the haem-access channel was also identified as a possible RB19-binding site (Figure 2A), the G169L variant was also obtained, whose leucine side chain completely blocks the haem-access channel, as shown in the crystal structure of a variant including the G169L mutation, compared with the WT DyP (Supplementary Figures S2B and S2A respectively).

The two sets of constants for oxidation of RB19, DMP and ABTS, and a single set for RB5 and VA, by the above five variants are shown in Table 1. The Y285F variant was also analysed, but no significant modification of the RB19 constants was observed. As a main conclusion, Trp-377 appears responsible for the high-turnover catalytic site, since its substitution (W377S variant) completely prevented high-turnover oxidation of RB19 and DMP (and decreased over 40-fold the catalytic efficiency of high-turnover ABTS oxidation). In contrast, similar Tyr-337 and Tyr-147 variants (and the double Y147F/Y337F variant) only slightly affected substrate oxidation, and did not remove the high-turnover site.

The above results are illustrated in Figures 7(A)–7(C), where kinetic plots for RB19 oxidation by WT DyP, and several tryptophan (W377S) and tyrosine (Y147S, Y337S and Y147F/Y337F) variants are shown respectively. It is observed that the DyP low-turnover site (Figure 7A, region *b*), remaining after Trp-377 removal (Figure 7B), does not correspond to Tyr-147 or Tyr-337, since changing these residues does not significantly affect the enzyme kinetics (Figure 7C). However, the disappearance of the low-turnover site in the G169L variant (Figure 7D, inset) suggests that this site involves the haem-access channel (although a decrease of the maximal turnover was also observed). Similar results were obtained for ABTS oxidation (Table 1), but the situation could be more complicated for DMP, suggesting that a third, still unidentified, site participates in oxidation of phenols by *A. auricula-judae* DyP. Finally, none of the mutations caused noticeable changes in oxidation of VA, which was highly inefficient in all cases, although Trp-377 appears to be the main residue involved.

### A global view of substrate oxidation sites in the *A. auricula-judae* and other basidiomycete DyPs

The enzyme kinetics reveal the existence of at least one high-turnover and one low-turnover oxidation site in *A. auricula-judae* DyP. Site-directed mutagenesis showed that high-turnover oxidation of RB19 and other substrates by DyP takes place at the same tryptophan residue (Trp-377), identified previously as forming a protein radical. This agrees with an 85–100% decrease in enzyme activity after modifying tryptophan residues with 0.2 mM NBS, and contrasts with the absence of a significant effect reported previously [9]. This discrepancy is probably due to the low NBS concentration used in the latter study. Modification of tyrosine residues with comparatively high TNM concentrations (40 mM) partially decreased (10–25%) the activity of DyP, as reported previously for the enzyme purified from a fungal culture [23]. However, this does not seem to be due to modification of Tyr-337 (or contiguous Tyr-147) since the enzyme activity was practically unaffected after site-directed mutagenesis of these two residues. A possibility suggested by favourable RB19 docking by PELE is low-turnover oxidation at the DyP haem channel. Interestingly, the *B. adusta* DyP has been crystallized with DMP occupying the entrance of a second channel near the haem propionates [7]. This channel does not exist in the *A. auricula-judae* DyP, where it is covered with a loop (residues 316–324). Therefore direct substrate oxidation by the haem cofactor in the latter DyP is only possible at the main haem access channel, in agreement with results from the G169L mutation that blocked the channel. A tyrosine residue could also be responsible for the second/third catalytic sites, in agreement with the partial activity loss after TNM modification. Nevertheless, once demonstrated that Trp-377 is the main substrate oxidation site in *A. auricula-judae* DyP, it is difficult to unambiguously identify these second/third catalytic sites due to the variety of exposed aromatic residues in this haem peroxidase.

The two aromatic residues forming the mixed tryptophanyl/tyrosyl radical in the H_2_O_2_-activated DyP of *A. auricula-judae* (Trp-377 and Tyr-337) are not a peculiarity of this enzyme, but they are significantly conserved among basidiomycete DyPs (Supplementary Figure S8). Among the 65 DyP sequences compared, 58 include a tryptophan residue homologous with Trp-377, and 48 include a tyrosine residue homologous with Tyr-337 (whereas the neighbour Tyr-147 is only conserved in 14 sequences). Therefore the presence of exposed aromatic residues able to form catalytic radicals appears as a common characteristic of basidiomycete, and probably other, DyPs. Although involvement of tyrosyl radicals in oxidation of bulky dyes by DyP has been compared recently with oxidation of recalcitrant aromatics by tryptophanyl radicals in ligninolytic classical peroxidases [4], the results of the present study suggest that tryptophanyl radicals are mainly responsible for substrate oxidation by these two phylogenetically unrelated peroxidase types.

## Online data

Supplementary data

Supplementary Movie S1. PELE trajectory for free substrate diffusion on DyP.
