# Supplementary material for: Catalytic surface radical in dye-decolorizing peroxidase: a computational, spectroscopic and site-directed mutagenesis study
Source: Biochem J. 2015 Feb 20;466(Pt 2):253–62. doi: 10.1042/BJ20141211 (PMC4357238; doi:10.1042/BJ20141211)
Supplement: Supplementary data [file bj4660253ntsadd.pdf]

SUPPLEMENTARY ONLINE DATA

Catalytic surface radical in dye-decolorizing peroxidase: A computational, spectroscopic and site-directed mutagenesis study

Dolores LINDE[*1], Rebecca POGNI[†1], Marina CAÑELLAS[‡§1], Fátima LUCAS[‡], Victor GUALLAR[‡¶], Maria Camilla BARATTO[†], Adalgisa SINICROPI[†], Verónica SÁEZ-JIMÉNEZ[*], Cristina COSCOLÍN[*], Antonio ROMERO[*], Francisco Javier MEDRANO[*], Francisco J. RUIZ-DUEÑAS[*2], and Angel T. MARTÍNEZ[*2]

[*]Centro de Investigaciones Biológicas, CSIC, Ramiro de Maeztu 9, E-28040 Madrid, Spain, [†]Department of Biotechnology, Chemistry and Pharmacy, University of Siena, I-53100, Siena, Italy, [‡]Joint BSC-CRG-IRB Research Program in Computational Biology, Barcelona Supercomputing Center, Jordi Girona 29, E-08034 Barcelona, Spain [§]Anaxomics Biotech, Balmes 89, E-08008 Barcelona, Spain and [¶]ICREA, Passeig Lluís Companys 23, E-08010 Barcelona, Spain

Supplementary methods for DyP production, activation and purification; supplementary results and discussion on electronic absorption and EPR spectra of WT DyP and haem pockets variants; three supplementary tables providing the crystallographic data (**Table S1**), spin density in pairwise comparison of Trp-377 and other residues (**Table S2**) and experimental and computed g-tensors and hfcc values for Tyr-337 radical species (**Table S3**); eight supplementary figures showing the chemical structures of the five DyP substrates assayed (**Fig. S1**), the haem access channel region in WT DyP and after G169L mutation (**Fig. S2**), the location of several tryptophan and tyrosine residues with respect to the haem cofactor (**Fig. S3**), the electronic absorption spectra of WT DyP (**Fig. S4**), the electronic absorption and EPR spectra of the D168N and R332L variants (**Fig. S5**), the optimal pH for DyP oxidation of different substrates (**Fig. S6**), kinetic and MALDI-TOF evidence for lack of a DyP activation mechanism (**Fig. S7**), Tyr-147, Tyr-337 and Trp-377 homolog residues in different clusters of basidiomycete DyP phylogram (**Fig. S8**), and conserved tryptophan and tyrosine residues in several basidiomycete DyPs (**Fig. S9**); one supplementary movie showing a PELE trajectory for RB19 free diffusion on DyP (**Movie S1**); and references for supplementary data are included below.

SUPPLEMENTARY METHODS

Chemicals

DNaseI, polymerase, and NBS were from Boehringer-Mannhein, Roche and Fluka, respectively. Bactopeptone and yeast extract were from Difco. *Nde*I and *Bam*HI were from New England Biolabs. Ampicillin, chloramphenicol, dithiothreitol, hemin, isopropyl-β-D-thiogalactopyranoside, lysozyme, sodium tartrate and TNM were from Sigma-Aldrich. Other chemicals were from Merck.

DyP production, activation and purification

E. coli BL21(DE3)pLysS cells with the pET23a-DyPI vector containing the *A. auricula-judae* mature WT DyP sequence (and those of DyP variants) were grown overnight at 37 ºC and 170 rpm in Luria Bertani broth (with 100 μg/mL of ampicillin and 34 μg/mL of chloramphenicol), and used to inoculate 2 L flasks containing 1 L of Terrific Broth (with ampicillin and chloramphenicol) that were grown for 3 h at 37 ºC and 200 rpm, induced with 1 mM isopropyl-β-D-thiogalactopyranoside, grown for further 4 h, and harvested by centrifugation.

---

[1]These authors contributed equally to this work

[2]To whom correspondence may be addressed: CIB, CSIC, Ramiro de Maeztu 9, E-28040 Madrid, Spain. Tel.: +34 918373112, Fax: +34 915360432 E-mail: ATMartinez@cib.csic.es and fjruiz@cib.csic.es.

The apoenzyme accumulated in inclusion bodies was solubilised in 50 mM Tris-HCl (pH 8.0) containing 8 M urea, 1 mM EDTA, and 1 mM dithiothreitol for 1 h at 4ºC. Subsequent "in vitro" activation was performed at 4 ºC using 0.2 M urea, 10 μM hemin, 0.02 mM dithiothreitol, 0.1 mM EDTA and 0.1 mg/ml of protein in 50 mM phosphate (pH 6). After 144 h, the folding mixture was concentrated, dialyzed against 20 mM sodium acetate (pH 4.3), and the insoluble material eliminated by centrifugation (13000 rpm, 30 min).

WT DyP and its site-directed variants were purified using a Resource Q column (GE Healthcare) coupled to an ÄKTA liquid chromatography system, using a gradient from 0 to 0.3 M of NaCl in 10 mM Tris-HCl (pH 7). DyPs were analyzed by SDS-PAGE to confirm the purity of the proteins. Absorption spectra were recorded in 10 mM sodium tartrate (pH 5) at 25 ºC in a Thermo Spectronic diode-array spectrophotometer. The DyP molar absorption coefficient (ε_{405} 117000 $M^{-1}\cdot cm^{-1}$) was calculated from (triplicate) Bradford determination of protein concentration, and used to estimate enzyme concentrations. For spectroscopic characterization of the transient states in the DyP catalytic cycle 1-10 eq of H_2O_2 were added to the resting enzyme in 10 mM sodium tartrate (pH 3 and 7).

Site-directed mutagenesis: Primers and PCR conditions

For PCR mutagenesis, direct and reverse primers designed complementary to opposite strands of the DNA region containing the desired mutation were used. The sequences of the direct primers (with the mutated codons in italics) used for mutagenic PCR was the following: Y147S or Y147F mutations 5′-CG TCG ATC TCG AAG CTC (*TCT* or *TTC*, respectively) TCG TTG TCG GCT TCG-3′; D168N mutation, 5′-G TTC GGC TTC CTT *AAC* GGA ATT GCT CAG CC-3′; G169L mutation, 5′-C GGC TTC CTT GAT *CTT* ATT GCT CAG CCC GC-3′; Y285F mutation, 5′-GG AAC AAC AAC TTC ACC *TTC* TCA CAC GCC G- 3′; Y337S or Y337F mutations, 5′-GGC ATC CCC (*TCT* or *TTC*, respectively) GGT CCT GAG GTT ACC-3′; and W377S mutation, 5′-C CTT CAG CAG ACG *TCT* GCC GAT AAC GCC AAC TTC CC-3′.

PCR reactions were carried out in an Eppendorf (Hamburg, Germany) Mastercycler Pro using 10 ng of template DNA, 250 μM each dNTP, 125 ng of direct and reverse primers, 2.5 units of polymerase (Expand Long Template PCR System), and the manufacture′s reaction buffer. Reaction conditions were as follows: i) a "hot start" of 95 ºC for 1 min; ii) 18 cycles at 95 ºC for 50 s, 55 ºC for 50 s, and 68 ºC for 10 min; and iii) a final cycle at 68 ºC for 10 min. The mutated sequences were confirmed by DNA sequencing using an ABI 3730 DNA Analyzer (Applied Biosystem).

Chemical modification

TNM in ethanol (0-40 mM and 0-30 mM for WT DyP and its W377S variant, respectively) or NBS (0-300 μM) were added to 3 μM enzyme in 50 mM Tris-HCl (pH 7) or 50 mM acetate (pH 4), respectively. The final concentration of ethanol in the TNM modification mixture was 2.6%. After 30 min of incubation at 25ºC, the samples were applied into a 30 kDa Centricon Millipore and diafiltrated with 3 vol of 10 mM tartrate (pH 5).

DyP crystallization, data collection and refinement

Crystallization trials with WT DyP and its site-directed variants (Y147S, D168N, W377S,Y147S/W377S and Y147S/G169L/W377S) were carried out by the sitting drop vapor diffusion method, in 96-well MRC2 plates with 50 μl reservoir solution, using the Emerald (Wizard screens I-III) and Jena Biosciences screenings (JBScreen kits 1-10) at 22 ºC. Drops consisted of 0.2 μl of protein solution (10 mg/ml in 10 mM sodium tartrate, pH 5.0) and 0.2 μl of reservoir solution. Crystals of WT DyP were obtained in 32.5% PEG 4000, and crystals of all the site-directed variants were obtained in PEG 2000 MME (30 to 35%).

The crystals were mounted in nylon loops and flash-frozen in liquid N_2 in the mother liquor using Paratone-N (Hampton Research) as cryoprotectant. X-ray diffraction intensities were collected (100 K) at the PROXIMA-1 beam line at SOLEIL (Gyf-sur-Yvette, France), and at XALOC beam line at

ALBA (Barcelona, Spain) using a Pilatus 6M detector. Data were indexed, integrated, merged and scaled using XDS and XSCALE [1].

The structure of DyP was solved by molecular replacement using the crystal structure of *B. adusta* DyP (PDB entry 4AFV) as the search model and the program AUTOMR of the PHENIX package [2]. The final model was obtained by successive refinement rounds (PHENIX package) followed by manual building with Coot [3] using σ_A weighted 2Fo-Fc and Fo-Fc electron density maps. Solvent molecules were introduced in the refinement, as implemented in the PHENIX package, and visually inspected. A total of 5% of reflections was used to calculate the R_{free} value throughout the refinement process. The structures of all the variants were solved using that of WT DyP as a model, and the final models were obtained in the same way as explained above. The structures were validated with MolProbity [4]. PyMOL [5] and Deep view/Swiss Pdb-Viewer (www.expasy.org/spdbv) were used for structure visualization and analysis, and image generation.

Enzyme kinetics

Steady-state kinetic constants of WT DyP and its site-directed variants on five selected substrates (whose structures are shown in **Fig. S1**) were determined using a Thermo Spectronic UV-visible spectrophotometer. For DMP, ABTS and VA oxidation, absorbance increases at 469 nm (dimeric coerulignone ε_{469}= 55,000 cm^{-1} M^{-1}), 436 nm (ABTS cation radical ε_{436}= 29,300 cm^{-1} M^{-1}) and 310 nm (veratraldehyde ε_{310}= 9,300 cm^{-1} M^{-1}) were followed, respectively. Absorbance decreases were followed in the cases of RB5 (ε_{598}= 30,000 cm^{-1} M^{-1}) and RB19 (ε_{595}= 10,000 cm^{-1} M^{-1}) oxidation resulting in dye decolorization. The ability to oxidize Mn^{2+} was investigated by the eventual formation of Mn^{3+}·tartrate complex (ε_{238} 6500 cm^{-1} M^{-1}) in 0.1 M tartrate (pH 5). Reactions were performed at 25 ºC in 100 mM tartrate, pH 3, except for VA and RB19 oxidation that pH 2.5 and 3.5 were used, respectively. All the substrates were tested using the enzymes at a final concentration of 10 nM (except VA whose oxidation was assayed with 100 nM enzyme) and 0.1 mM H_2O_2. Apparent affinity constant (Michaelis–Menten constant, K_m), turnover number (catalytic constant, k_{cat}) and their standard errors were obtained by non-linear least-squares fitting of the experimental measurements to the Michaelis-Menten model. The catalytic efficiency (k_{cat}/K_m) values with their standard errors were calculated fitting the experimental data to the normalized Michaelis-Menten equation: $v= (k_{cat}/K_m)[S]/(1+[S]/K_m)$. The catalytic efficiency constant for VA was obtained by fitting data to a linear regression equation ($k_{cat}= (k_{cat}/K_m)[S]$) since no enzyme saturation was attained.

MALDI-TOF after steady-state turnover

Eventual changes in the molecular mass of WT DyP, similar to those produced during activation of the *T. cervina* LiP by formation of a VA adduct with the catalytic tyrosine residue, were analyzed by MALDI-TOF using an Autoflex III instruments, and 2,5-dihydroxyacetophenone matrix, after enzyme reaction with H_2O_2 and VA, as previously described [6]. For these analyses, 10 μM enzyme was incubated for 1 h at 25 °C, in sodium tartrate (pH 3) containing 0.5 mM H_2O_2 and 10 mM VA, and compared with a control without H_2O_2 and VA. The reaction mixture was centrifuged into a 30 kDa Centricon Millipore, diafiltrated with 3 vol of 20 mM tartrate (pH 5).

Computational analyses: PELE, MD and QM/MM calculations

The model used is based on the recombinant *A. auricula-judae* DyP crystal structure solved here. As the optimum pH for oxidation of RB19 by DyP is 3.5 we prepared the starting structure accordingly. All ionizable residues were inspected with Schrodinger's protein preparation wizard [7] and with the H++ web server [8]. His-304 was assessed to be δ-protonated, His-115 ε-protonated, and the remaining are double protonated (positively charged). At this low pH several aspartic (residues 8, 12, 84, 129, 189, 246 and 270) and glutamic (residues 158, 220, 225 and 432) acids are in their acidic form, while all other ones are found in their anionic states. Electrostatic potential atomic charges on the RB19 substrate (**Fig. S1A**), to be used in PELE and molecular

dynamics (MD), were obtained from an optimization with Jaguar [9] at the DFT/M06-L level with the 6-31G** basis set and a PB implicit solvent.

Once the initial protein structure was prepared, RB19 was placed manually in 20 initial random positions on the protein's surface and the protein-ligand conformational space was explored with PELE [10]. This is a Monte Carlo based algorithm that produces new configurations through a sequential ligand and protein perturbation, side chain prediction and minimization steps. New configurations are then filtered with a Metropolis acceptance test, where the energy is described with an all-atom OPLS force field and a surface generalized Born solvent. In this way it is possible to locate and characterize local and global minima structures for the most favorable protein-ligand interactions. Results shown are based on 160 independent 48-h PELE simulations. Enhanced local sampling on Trp-377 surface site was obtained with a 5 ns MD simulation with DESMOND [11]. Such analysis allowed us to investigate the effect of solvent and charge fluctuations on the oxidative tendency of Trp-377 and RB19.

QM/MM calculations were performed with Qsite [12]. This method allows for the incorporation of the complete protein structure (as well as solvent and ions) at the atomic level while electronic structure based methods are employed in a sub-section of the system. Most calculations were performed at the M06-L(lacvp*)/OPLS level. Spin densities for the highly reactive compound I were computed at the quartet spin state (easier to converge than the isoenergetic doublet one). Spin density on residues was computed by adding all the atomic spin density contributions. Those residues included in the QM region with/without the haem cofactor (compound I) and a substrate (RB19) molecule are indicated in each case. Additionally, electron transfer pathway calculations were performed with the QM/MM e-pathway approach (using the Hartee-Fock method) [13]. This approach maps those residues with higher probability to participate in the electron transfer pathway by finding, iteratively, those regions of the transfer region with lower ionization energy. In particular, we computed the pathway from Trp377 by considering in the QM region His-304, Ile-305, Arg-306, Lys-307, Thr-308, Arg-309, Pro-310, Arg-311, Leu-323, Ser-324, Ala-325, Leu-373, Gln-374, Gln-375 and Asp-395.

EPR equipments

CW X-band (9.8 GHz) measurements were performed with a Bruker E500 Elexsys Series using the Bruker ER 4122SHQE cavity, equipped with an Oxford helium continuous flow cryostat (ESR900). W-band (94.17 GHz) experiments were recorded on a Bruker Elexsys E600 spectrometer, operating in continuous wave, equipped with a 6T split-coils superconducting magnet (Oxford Instrument), using a continuous helium flow cryostat (Oxford Instrument).

SUPPLEMENTARY RESULTS AND DISCUSSION

Haem pocket site-directed variants: Electronic absorption and EPR spectra

Asp-168 in DyP occupies the position of conserved distal histidine in classical peroxidases. This residue and the neighbor Arg-332 are expected to play a central role in catalysis since both the D168N and the R332L variants obtained were fully inactive on the different substrates.

The electronic absorption spectra of DyP resting and H_2O_2-activated (2 eq) states at pH 3 (the optimal pH for DyP activity, as shown in **Fig. S6**) are included in **Fig. S4A**. The latter exhibit the main peak at 403 nm, and small maxima at 529, 558, 597 and 620 nm, all of them being characteristic for compound I. However, the D168N and R332L variants were unable to react with H_2O_2 (pH 3) as shown by their unchanged absorption spectra (**Fig. S5A** and **B**). When 2 eq of H_2O_2 were added to the resting enzyme at pH 7 (which showed reduced absorbance at 406 and 630 nm and the 506 nm maximum displaced to 502 nm with respect to the pH 3 resting state) a compound II-like spectrum, with main peak at 418 nm and small maxima at 530, 554 and 634 nm, was obtained (**Fig. S4B**). Unexpectedly, no compound II-like spectrum could be obtained at pH 3 by self-reduction or using other H_2O_2 concentrations.

The involvement of Asp-168 and Arg-332 in DyP reactivity with H_2O_2, suggested by the electronic absorption spectra, was investigated by EPR. The resting state spectrum of the D168N variant showed a ferric iron in a rhombic environment (g_{xx}= 6.06, g_{yy}=5.6 and g_{zz}= 2.0) (**Fig. S5C**, black trace). After H_2O_2 activation at pH 3 (**Fig. S5C**, red trace) only a small decrease in the ferric iron signal is recorded and no protein radicals are formed. An almost undetectable amount of compound I in the form of the porphyrin radical can be identified superimposed to the ferric g= 2 region of the EPR spectrum (**Fig. S5C**, inset). The results were similar for the R332L variant (**Fig. S5C**), although a small radical signal was observed (4% radical yield for R332L at pH 3 compared with 58% for WT DyP, under the same experimental conditions) (**Fig. S5D**, inset).

The similarities in the haem pocket architecture of unrelated DyP and classical (plant-fungal-prokaryotic) peroxidases would result from adaptative convergence. This is the case of: i) the conserved proximal histidine (*A. auricula-judae* DyP His-304) acting as the fifth haem iron ligand in both enzyme types; and ii) the opposite haem side residues (DyP Asp-168 and Arg-332) most probably contributing to reaction with H_2O_2, as distal histidine and arginine do in classical peroxidases. The latter was demonstrated by the loss of DyP ability to form compound I after the D168N and R332L mutations (as shown by electronic absorption and EPR spectra). A central role of the conserved aspartic acid or arginine in H_2O_2 reaction have been claimed in the *B. adusta* [14] and *R. jostii* DyP [15], respectively, but the present results show that both residues are required for *A. auricula*-judae DyP activity, although some protein radical signal was still observed in the EPR spectrum of the R332L variant. As reported for other DyPs [14], no compound II spectrum was observed during self-reduction of the *A. auricula-judae* DyP (at physiological pH 3), a fact that suggests fastest self-reduction of compound II than of compound I, in contrast with other fungal peroxidases [16]. At the other side of the haem, Asp-395, also conserved in other DyPs [14], would help proximal His-304 to modulate the haem electron-deficiency, as the proximal aspartic acid in classical peroxidases [17].

SUPPLEMENTARY TABLES

Table S1 Data collection and refinement statistics of *A. auricula-judae* WT DyP expressed in *E. coli* and five site-directed variants

	WT DyP	Y147S	D168N	W377S	Y147S/W377S	Y147S/G169L/ W377S
Data collection						
Space group	C2	C2	C2	C2	C2	C2
Cell constants:						
-a (Å)	154.2	184.0	183.7	184.0	154.2	104.4
-b (Å)	100.5	55.9	56.3	56.2	100.5	56.1
-c (Å)	128.6	104.0	103.8	104.1	128.6	82.0
-β (º)	123.7	118.3	117.8	118.0	123.7	96.9
Resolution range (Å)	50.00-1.79 (1.89-1.79)[a]	50.00-1.05 (1.11-1.05)	50.00-1.05 (1.11-1.05)	50.00-1.15 (1.22-1.15)	50.00-1.40 (1.49-1.40)	50.00-1.20 (1.27-1.20)
Total reflections	620794	2174931	2352392	1894778	1142801	949627
Unique reflections	150630	364516	405267	319222	181153	147432
R_{merge} (%)	13.4 (99.5)	8.8 (105.2)	6.8 (50.5)	6.6 (38.4)	7.0 (97.2)	6.0 (45.8)
CC1/2	99.7 (70.5)	99.9 (45.3)	99.9 (82.3)	99.8 (87.3)	99.9 (71.1)	99.9 (93.8)
Completeness (%)	97.1 (90.3)	82.6 (29.8)	91.5 (54.3)	95.2 (72.1)	99.3 (96.8)	99.2 (95.4)
$\langle I/\sigma(I)\rangle$	6.59 (1.03)	12.3 (1.0)	12.7 (1.7)	13.1 (2.3)	14.2 (1.5)	15.4 (3.1)
Multiplicity	4.1 (3.5)	6.0 (2.9)	5.8 (3.3)	5.9 (3.5)	6.3 (5.2)	6.4 (6.1)
Subunits/asymmetric unit	4	2	2	2	2	1
Wilson B factor ($Å^2$)	21.52	10.27	8.91	15.35	23.44	12.07
Refinement						
Resolution range	50.00-1.79	50.00-1.05	50.00-1.05	50.00-1.15	50.00-1.79	50.00-1.20
Working reflections	150153	364071	405142	319118	150153	146680
R_{work}/R_{free}	0.193/0.240	0.135/0.157	0.131/0.150	0.131/0.150	0.144/0.184	0.139/0.158
Protein atoms (non H)	13217	6705	6721	6586	6663	3302
Haem group	172	94	94	86	86	43
Water molecules	1564	1582	1887	1156	1489	700
Mean B factors ($Å^2$):						
-Protein atoms (non H)	24.83	11.86	10.46	17.16	12.65	15.04
-Haem group	19.66	7.90	6.97	10.99	9.27	10.07
-Water molecules	30.29	24.91	23.35	29.60	24.58	27.80
Deviations from ideality:						
-rmsd bond lengths (Å)	0.009	0.009	0.010	0.004	0.009	0.007
-rmsd angles (º)	1.210	1.410	1.432	0.930	1.210	1.257
Ramachandran statistics:						
-Preferred (%)	97.86	98.54	98.66	98.76	98.42	98.87
-Allowed (%)	2.03	1.46	1.23	1.20	1.58	1.13
-Outliers (%)	0.11	0.00	0.11	0.00	0.00	0.00
PDB code	4W7J	4W7K	4W7L	4W7M	4W7N	4W7O

[a]Data in parenthesis correspond to the last resolution layer.

Table S2 Spin density in pairwise QM/MM comparison of Trp-377 and other surface residues

Pairwise comparisons of spin density distribution in Trp-377 and other surface residues at different distances to haem of *A. auricula-judae* DyP were performed by QM/MM calculations (the distances, in parentheses, are between the residue gamma carbon and the haem iron).

Residue 1	Spin (%)	Residue 2	Spin (%)
Trp-377 (12.5 Å)	100	Trp-105 (19.5 Å)	0
Trp-377	38	Trp-207 (32.0 Å)	62
Trp-377	100	Tyr-285 (14.5 Å)	0
Trp-377	100	Tyr-337 (15.9 Å)	0

Table S3 Comparison of experimental (from W377S variant) and computed (for DyP Tyr-337) tyrosine radical EPR parameters

Experimental (W377S variant) and B3LYP/EPR-II g-tensors (g_i) and hfcc values (A_i, in MHz) computed at the B3LYP/AMBER optimized geometries for Tyr-337 radical species are shown.

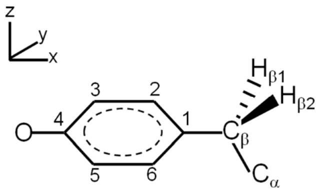

			$^a g_i$	$A_i(H_{\beta 1})$	$A_i(H_{\beta 2})$	$A_i(H_2)$	$A_i(H_6)$	$A_i(H_3)$	$A_i(H_5)$
W377S		x	2.0075	16.6	15.4	2.0	2.7	26.0	24.0
		y	2.0043	17.3	16.0	4.5	5.2	21.0	19.0
		z	2.0020	22.4	21.0	8.1	9.0	7.0	7.0
		Iso	2.0046	18.8	17.5	4.9	5.6	18.0	16.6
B3LYP/EPR-II[b]	Tyr-337$^\bullet$	x	2.0066	16.6	15.4	2.0	2.7	-26.3	-24.2
		y	2.0044	17.3	16.1	4.5	5.2	-20.3	-18.7
		z	2.0022	22.4	21.5	8.1	9.0	-6.6	-5.7
		Iso	2.0044	18.8	17.7	4.9	5.6	-17.8	-16.2

[a]The errors on the experimental g values are: $\pm$ 0.0001 for g_x, $\pm$ 0.0002 for g_y and g_z; [b]The computed hfcc values for Tyr-337 radical have been used to simulate the experimental EPR spectrum of the radical species in the W377S variant. Although the B3LYP/EPRII g_x value differs from the experimental one, the computed values clearly indicate that the tyrosine radical is embedded in a strongly H-bonded environment, which is in accord with the experiments.

SUPPLEMENTARY FIGURES

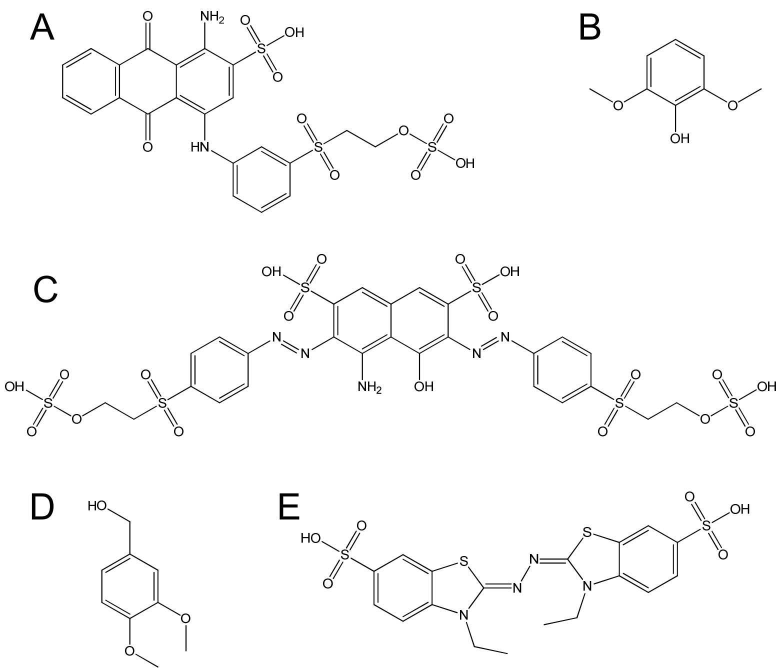

Figure S1 Chemical structure of the five DyP substrates assayed

(A) RB19 (1-amino-9,10-dioxo-4-((3-((2-(sulfooxy)ethyl)sulfonyl)phenyl)amino)-9,10-dihydroanthracene-2-sulfonic acid). **(B)** 2,6-Dimeth-oxyphenol. **(C)** RB5 (4-amino-5-hydroxy-3,6-bis((E)-(4-((2-(sulfooxy)ethyl)sulfonyl)phenyl)-diazenyl)-naphthalene-2,7-disulfonic acid). **(D)** Veratryl (3,4-dimethoxybenzyl) alcohol. **(E)** ABTS (2,2'-azino-bis(3-ethylbenzothiazoline-6-sulphonic acid).

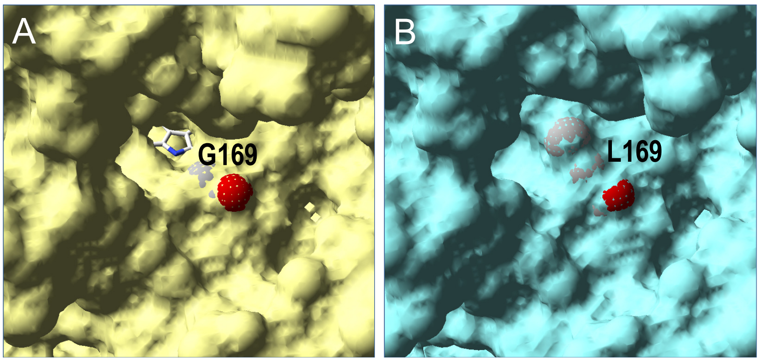

Figure S2 Haem access channel region in WT DyP and after G169L mutation

(A) Solvent access surface in the WT DyP crystal structure (PDB 4W7J) showing channel accessing to the haem cofactor (CPK sticks) and the position of Gly-169 (CPK spheres). **(B)** Solvent access surface in the C169L/Y146S/W377S variant (PDB 4W7O) showing the blocked haem access by the Leu-169 (CPK spheres) side-chain.

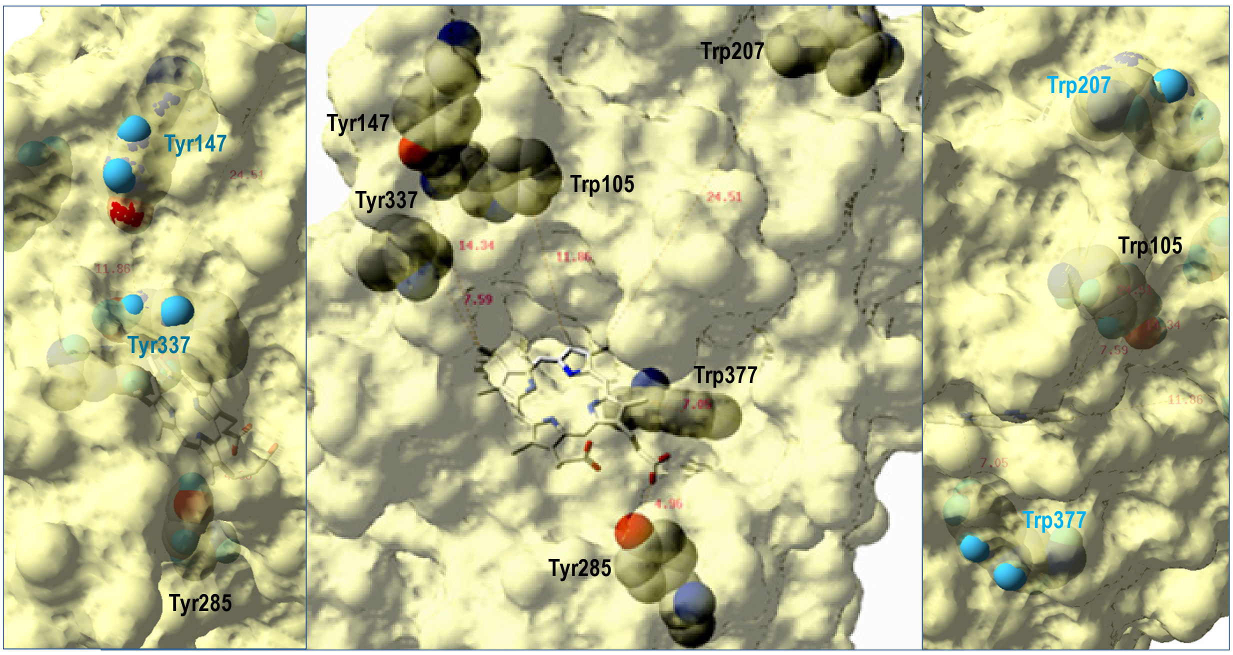

Figure S3 Location of several tryptophan and tyrosine residues near the haem cofactor of DyP. ***(center)*** Semitransparent DyP surface showing the channel accessing the haem cofactor (CPK sticks) and the position of Trp-105, Tyr-147, Trp-207, Tyr-285, Tyr-337 and Trp-377 (CPK spheres) at 12, 14, 25, 5, 8 and 7 Å from the cofactor, respectively. ***(left*** and ***right)*** Exposed Tyr-147, Trp-207, Tyr-337 and Trp-377, and buried Trp-105 and Tyr-285 (CPK spheres with H atoms included). From PDB 4W7J.

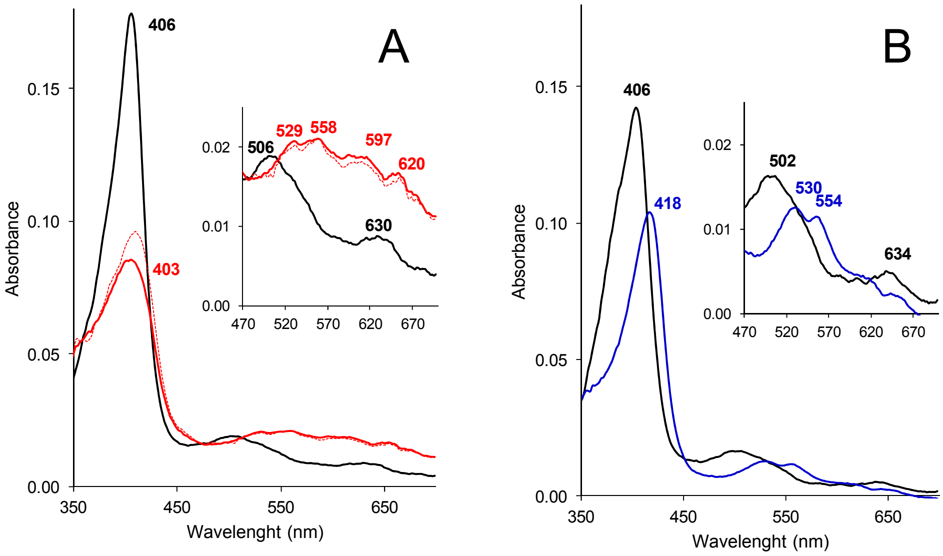

Figure S4 Electronic absorption spectra of DyP

(A) Compound I spectrum (red) obtained 2 s after addition of 2 eq of H_2O_2 to DyP resting state (black) in 10 mM tartrate, pH 3, showing the main Soret band at 403 and 406 nm, respectively, and small charge transfer bands in the inset (dotted line shows the spectrum obtained 0.02 s after H_2O_2 addition). **(B)** Compound II-like spectrum (blue) obtained 2 s after addition of 2 eq of H_2O_2 to DyP resting state (black) in 10 mM Tris-HCl, pH 7, showing the main Soret band at 418 and 406 nm, respectively, and small charge transfer bands in the inset. Spectra were obtained using a stopped-flow rapid spectrophotometer (Bio-Logic) equipped with three-syringe module (SFM300), diode array detector (J&M), and Bio-Kine software.

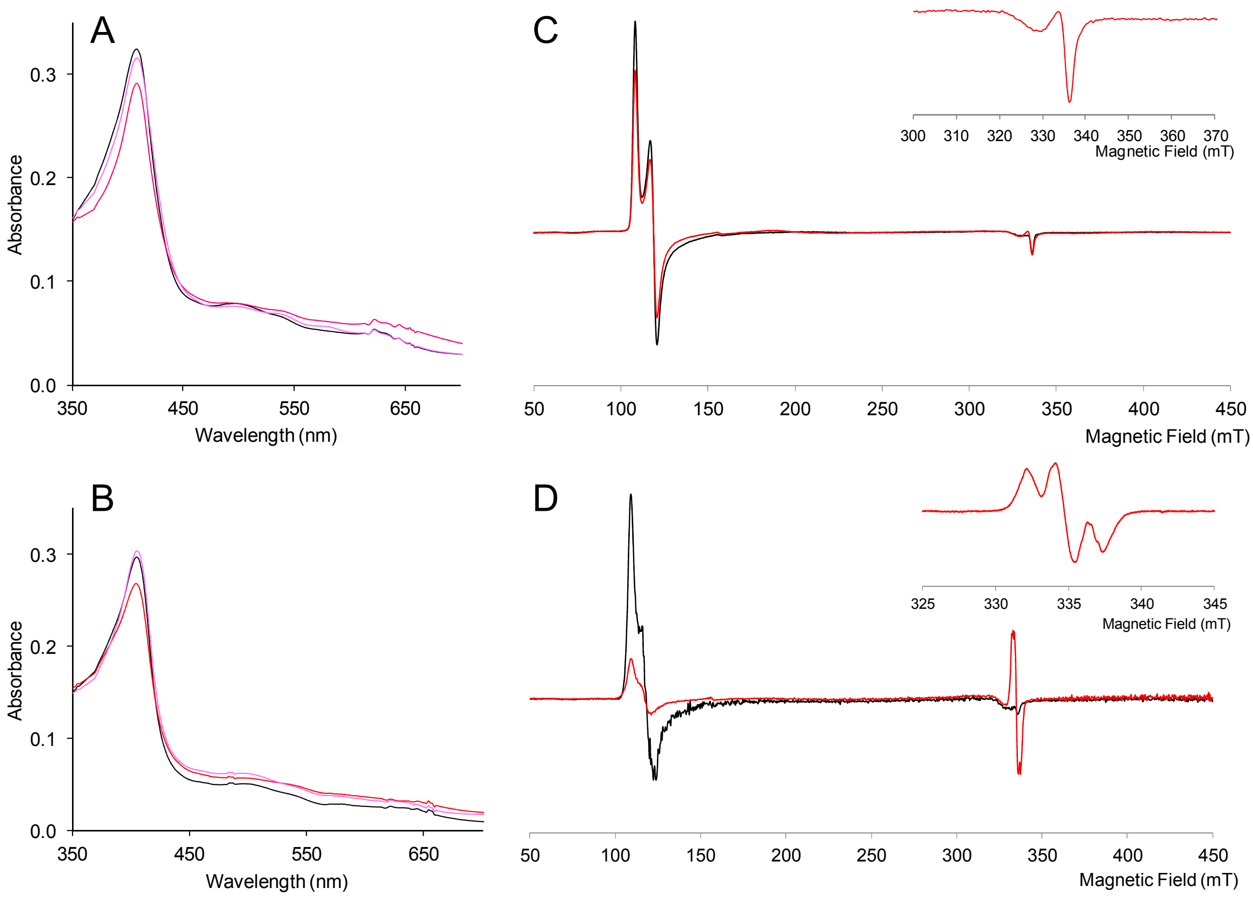

Figure S5 Electronic absorption and EPR spectra of peroxide-activated D168N and R332L variants

(A and **B)** Electronic absorption spectra of 10 µM D168N and R332L variants 0 (black), 50 (red) and 1600 (pink) s after addition of 10 eq of H_2O_2 in 10 mM tartrate, pH 3, respectively. **(C** and **D)** X-band EPR spectra of the D168N and R332L variants (160 µM) before (black) and after (red) addition of 10 eq of H_2O_2 in 70 mM tartrate, pH 3, respectively (insets, detail of the g= 2 region). Experimental conditions: ν= 9.39 GHz, 2 mW microwave power, 0.4 mT modulation amplitude)

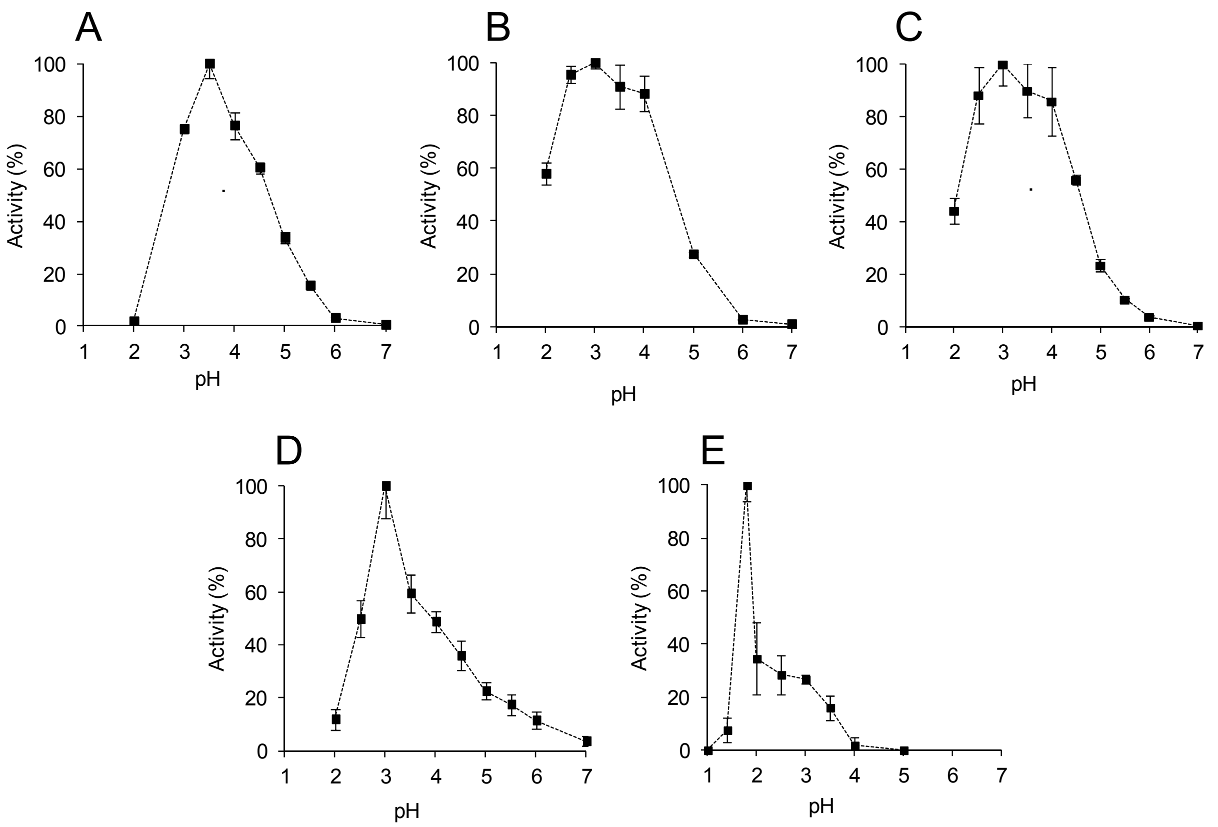

Figure S6 Effect of pH for *A. auricula-judae* DyP oxidation of different substrates

(A-E) Relative activity of recombinant DyP on RB19 (50 µM), DMP (0.5 mM), ABTS (0.5 mM), RB5 (25 µM) and VA (10 mM), respectively, measured in 50 mM Britton-Robinson buffer (pH 2-12) and KCl-HCl (pH 1) buffers. Bars represent the standard deviations of the means of three measurements

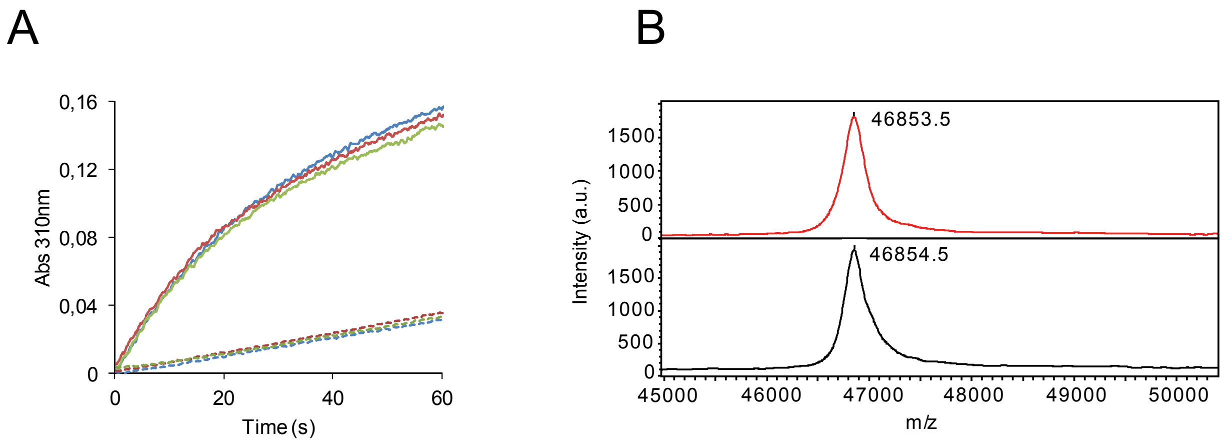

Figure S7 Kinetic and MALDI-TOF evidence for lack of a tyrosine activation mechanism in DyP

(**A**) Absorbance changes at 310 nm (veratraldehyde formation) during oxidation of VA by 10 µM WT DyP (continuous line) and W377S variant (dotted line) previously treated (1 h at 25 ºC) with VA (10 mM) in the presence of 20 µM (red trace) and 100 µM (green trace) H_2O_2, compared with a control incubated with VA alone (blue trace) (reactions in 0.1 M tartrate, pH 2.5, containing 2.5 mM VA, 0.1 mM H_2O_2, and 0.1 mM enzyme). **(B)** MALDI-TOF analysis of the WT DyP (10 µM) treated with 10 mM VA and 0.5 mM H_2O_2 (black profile) compared with an enzyme control incubated without VA and H_2O_2 (red profile). The similar results obtained confirm the absence of a covalent enzyme-VA adduct for activation of a DyP tyrosine residue, as found in *T. cervina* LiP [6].

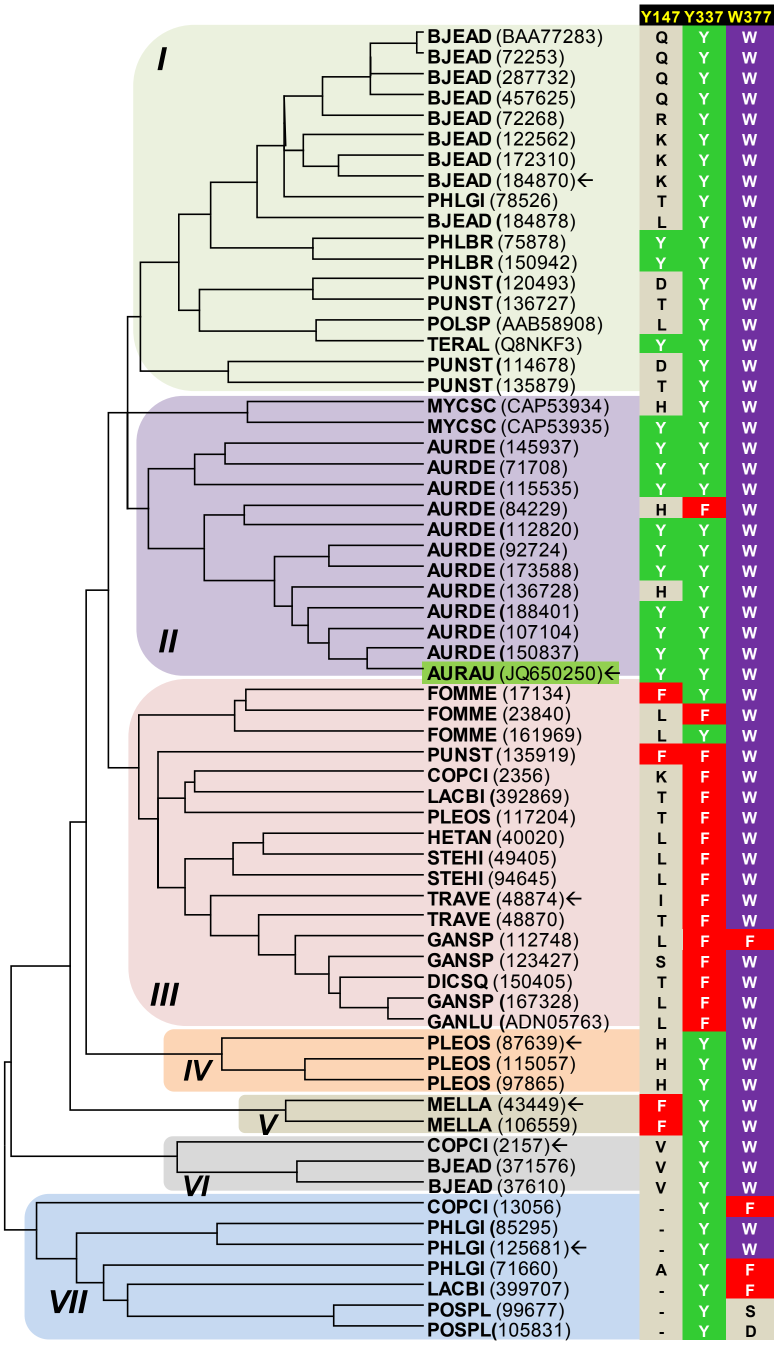

Figure S8 Tyr-147, Tyr-337 and Trp-377 homolog residues in different clusters of basidiomycete DyP phylogram

Sixty-five sequences of basidiomycete DyPs were obtained from the genomes of *Auricularia delicata* (AURAU), *Bjerkandera adusta* (BJEAD), *Coprinopsis cinerea* (COPCI), *Dichomitus squalens* (DICSQ), *Fomitiporia mediterranea* (FOMME), *Ganoderma* sp (GANSP), *Heterobasidion annosum* (HETAN), *Laccaria bicolor* (LACBI), *Melampsora laricis-populina* (MELLA), *Phlebia brevispora* (PHLBR), *Phlebiopsis gigantea* (PHLGI), *Pleurotus ostreatus* (PLEOS), *Postia placenta* (POSPL), *Punctularia strigosozonata* (PUNST), *Stereum hirsutum* (STEHI), and *Trametes versicolor* (TRAVE) available at the JGI (http://genome.jgi.doe.gov) together with GenBank sequences of *A. auricula-judae* (AURAU), *Ganoderma lucidum* (GANLU), *Mycetinis scorodonius* (MYCSC) and *Termitomyces albuminosus* (TERAL). The phylogram obtained with MEGA5 [18] includes three main clusters (I-III and VII; cluster-II containing the *A. auricula-judae* DyP) and three small ones (IV-VI). The JGI and GenBank references are provided, together with indication of the residues occupying the positions homologous to those of *A. auricula-judae* Tyr-147, Tyr-337 and Trp-377 in each of the sequences (details of homology models for the seven DyPs marked with arrows, as representative of the different clusters, are shown in **Fig. S9**).

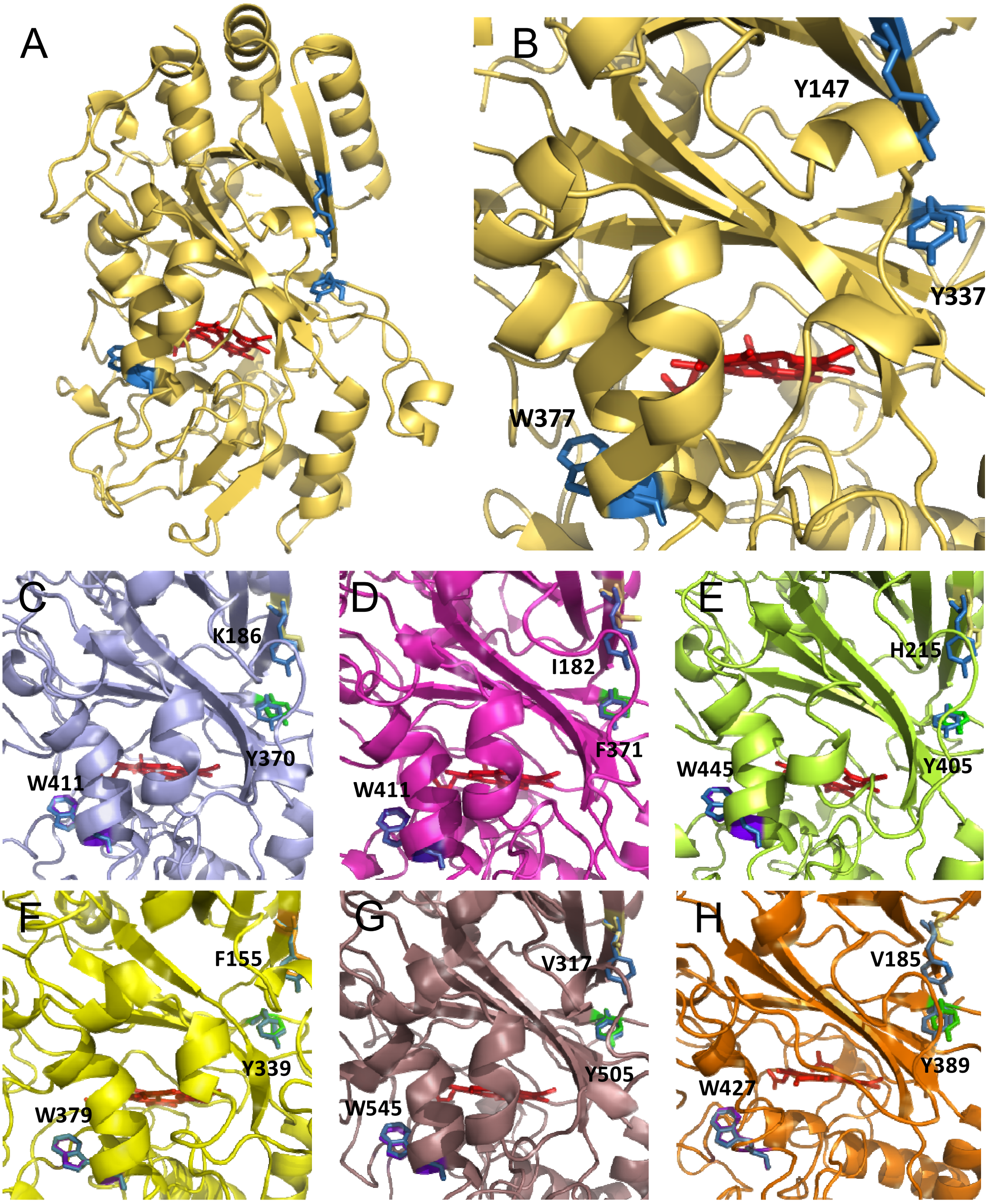

Figure S9 Residues homologous of *A. auricula-judae* DyP Trp147, Tyr337 and Trp377 in basidiomycete DyPs representative for the different clusters of Fig. S8

(A and **B)** Scheme of the whole crystal structure of recombinant *A. auricula-judae* DyP (4W7J) and detail of Tyr-147, Tyr-337 and Trp-377 near the haem cofactor, respectively. **(C-H)** Detail of residues homologous to the above *A. auricula-judae* DyP residues in molecular models for DyPs from the sequenced genomes of *B. adusta* (JGI 184870), *T. versicolor* (JGI 48874), *P. ostreatus* (JGI 87639), *M. laricis-populina* (JGI43449), *C. cinerea* (JGI 2157) and *P. gigantea* (JGI 125681), respectively.

SUPPLEMENTARY MOVIE

Movie S1 PELE trajectory for free substrate diffusion on DyP

One of the 160 free (non-biased) simulations for RB19 diffusion on the DyP molecule provided by PELE software is included, showing substrate binding at the haem access channel and near Trp-377.

REFERENCES FOR SUPPLEMENTARY DATA

1 Kabsch, W. (2010) XDS. Acta Crystallographica Section D-Biological Crystallography, **66,** 125-132

2 Adams, P. D., Afonine, P. V., Bunkoczi, G., Chen, V. B., Davis, I. W., Echols, N., Headd, J. J., Hung, L. W., Kapral, G. J., Grosse-Kunstleve, R. W., Mccoy, A. J., Moriarty, N. W., Oeffner, R., Read, R. J., Richardson, D. C., Richardson, J. S., Terwilliger, T. C. and Zwart, P. H. (2010) PHENIX: a comprehensive Python-based system for macromolecular structure solution. Acta Crystallographica Section D-Biological Crystallography, **66,** 213-221

3 Emsley, P. and Cowtan, K. (2004) Coot: model-building tools for molecular graphics. Acta Crystallogr. D. Biol. Crystallogr., **60,** 2132

4 Davis, I. W., Leaver-Fay, A., Chen, V. B., Block, J. N., Kapral, G. J., Wang, X., Murray, L. W., Arendall III, W. B., Snoeyink, J., Richardson, J. S. and Richardson, D. C. (2007) MolProbity: all-atom contacts and structure validation for proteins and nucleic acids. Nucleic Acids Res., **35,** W375-W385

5 de Lano, W. L. (2002) The PyMOLl molecular graphics system, DeLano Scientific, San Carlos, CA, USA

6 Miki, Y., Pogni, R., Acebes, S., Lucas, F., Fernández-Fueyo, E., Baratto, M. C., Fernández, M. I., de los Ríos, V., Ruiz-Dueñas, F. J., Sinicropi, A., Basosi, R., Hammel, K. E., Guallar, V. and Martínez, A. T. (2013) Formation of a tyrosine adduct involved in lignin degradation by *Trametopsis cervina* lignin peroxidase: A novel peroxidase activation mechanism. Biochem. J., **452,** 575-584

7 Sastry, G. M., Adzhigirey, M., Day, T., Annabhimoju, R. and Sherman, W. (2013) Protein and ligand preparation: parameters, protocols, and influence on virtual screening enrichments. Journal of Computer-Aided Molecular Design, **27,** 221-234

8 Anandakrishnan, R., Aguilar, B. and Onufriev, A. V. (2012) H++3.0: automating pK prediction and the preparation of biomolecular structures for atomistic molecular modeling and simulations. Nucleic Acids Res., **40,** W537-W541

9 Schrödinger. (2011) Jaguar 7.8, LCC, New York

10 Borrelli, K. W., Vitalis, A., Alcantara, R. and Guallar, V. (2005) PELE: Protein energy landscape exploration. A novel Monte Carlo based technique. J. Chem. Theory Comput., **1,** 1304-1311

11 Bowers, K. J., Chow, E., Xu, H., Dror, R. O., Eastwood, M. P., Gregersen, B. A., Klepeis, J. L., Kolossváry, I., Moraes, M. A., Sacerdoti, F. D., Salmon, J. K., Shan, Y. and Shaw, D. E. (2006) Scalable algorithms for molecular dynamics simulations on commodity clusters. Proc. ACM/IEEE Conference on Supercomputing (SC06), Tampa, 11-17 November, 43

12 Schrödinger. (2011) QSite 5.7, LCC, New York

13 Guallar, V. and Wallrapp, F. (2008) Mapping protein electron transfer pathways with QM/MM methods. J. R. Soc. Interface, **5,** S233-S239

14 Sugano, Y., Muramatsu, R., Ichiyanagi, A., Sato, T. and Shoda, M. (2007) DyP, a unique dye-decolorizing peroxidase, represents a novel heme peroxidase family. Asp171 replaces the distal histidine of classical peroxidases. J. Biol. Chem., **282,** 36652-36658

15 Singh, R., Grigg, J. C., Armstrong, Z., Murphy, M. E. P. and Eltis, L. D. (2012) Distal heme pocket residues of B-type dye-decolorizing peroxidase: Arginine but not aspartate is essential for peroxidase activity. J. Biol. Chem., **287,** 10623-10630

16 Pérez-Boada, M., Ruiz-Dueñas, F. J., Pogni, R., Basosi, R., Choinowski, T., Martínez, M. J., Piontek, K. and Martínez, A. T. (2005) Versatile peroxidase oxidation of high redox potential aromatic compounds: Site-directed mutagenesis, spectroscopic and crystallographic investigations of three long-range electron transfer pathways. J. Mol. Biol., **354,** 385-402

17 Martínez, A. T. (2002) Molecular biology and structure-function of lignin-degrading heme peroxidases. Enzyme Microb. Technol., **30,** 425-444

18 Tamura, K., Peterson, D., Peterson, N., Stecher, G., Nei, M. and Kumar, S. (2011) MEGA5: Molecular evolutionary genetics analysis using maximum likelihood, evolutionary distance, and maximum parsimony methods. Mol. Biol. Evol., **28,** 2731-2739
